# A Calmodulin-Like Gene (*GbCML7*) for Fiber Strength and Yield Improvement Identified by Resequencing Core Accessions of a Pedigree in *Gossypium barbadense*

**DOI:** 10.3389/fpls.2021.815648

**Published:** 2022-02-03

**Authors:** Nan Zhao, Weiran Wang, Kaiyun Jiang, Corrinne E. Grover, Cheng Cheng, Zhuanxia Pan, Cunpeng Zhao, Jiahui Zhu, Dan Li, Meng Wang, Li Xiao, Jing Yang, Xinmin Ning, Bin Li, Haijiang Xu, Ying Su, Alifu Aierxi, Pengbo Li, Baosheng Guo, Jonathan F. Wendel, Jie Kong, Jinping Hua

**Affiliations:** ^1^Joint Laboratory for International Cooperation in Crop Molecular Breeding, Ministry of Education/College of Agronomy and Biotechnology, China Agricultural University, Beijing, China; ^2^Institute of Economic Crops, Xinjiang Academy of Agricultural Sciences, Ürümqi, China; ^3^Department of Ecology, Evolution and Organismal Biology, Iowa State University, Ames, IA, United States; ^4^Institute of Cotton Research, Shanxi Agricultural University, Yuncheng, China; ^5^Cotton Research Institute, Hebei Academy of Agriculture and Forestry Sciences, Shijiazhuang, China

**Keywords:** pedigree breeding, fiber strength, haplotype interaction, joint improvement of quality and yield, *Gossypium barbadense*

## Abstract

Sea Island cotton (*Gossypium barbadense*) is world-renowned for its superior natural fiber. Although fiber strength is one of the most important fiber quality traits, genes contributing to fiber strength are poorly understood. Production of sea island cotton also is inextricably linked to improving its relatively low yield, thus enhancing the importance of joint improvement of both fiber quality and yield. We used genomic variation to uncover the genetic evidence of trait improvement resulting from pedigree breeding of Sea Island cotton. This pedigree was aimed at improving fiber strength and yielded an elite cultivar, XH35. Using a combination of genome-wide association study (GWAS) and selection screens, we detected 82 putative fiber-strength-related genes. Expression analysis confirmed a calmodulin-like gene, *GbCML7*, which enhanced fiber strength in a specific haplotype. This gene is a major-effect gene, which interacts with a minor-effect gene, *GbTUA3*, facilitating the enhancement of fiber strength in a synergistic fashion. Moreover, *GbCML7* participates in the cooperative improvement of fiber strength, fiber length, and fiber uniformity, though a slight compromise exists between the first two of these traits and the latter. Importantly, *GbCML7* is shown to boost yield in some backgrounds by increasing multiple yield components to varying degrees, especially boll number. Our work provides valuable genomic evidence and a key genetic factor for the joint improvement of fiber quality and yield in Sea Island cotton.

## Introduction

Cotton (*Gossypium* spp.) is a principal economic crop and important strategic reserve material, mainly due to its natural fiber (Su et al., 2020). Sea island cotton (*Gossypium barbadense*) is well-known for its excellent fiber quality (length, fitness, and strength) (Yu et al., 2013; Shi Y. Z. et al., 2015; Wang et al., 2018); however, the yield from this species typically is low, and thus *G. barbadense* accounts for only about 2% of the world cotton output (Kong, 2002). While Xinjiang is the main sea island cotton planting area in China, this region accounts for approximately 30% of the total sea island cotton production worldwide (Alifu et al., 2020; Yu et al., 2021). As of 2018, 68 elite sea island cotton cultivars were bred in Xinjiang since 1953 (Kong et al., 2018). This history provides the opportunity to explore the genetic factors underlying the improvement of fiber quality and yield during the breeding process of Xinjiang Sea Island cotton.

Molecular genetic studies of sea island cotton have become more sophisticated with the application of genome sequencing and assembly technologies. In 2015, the first sea island cotton genome (lines 3–79) was sequenced successfully, opening a new chapter of sea island cotton genomic analyses (Yuan et al., 2015). Since that time, 3–79 was the subject of additional sequencing and/or genome updates (Wang et al., 2018; Chen et al., 2020), while genomes for additional sea island cotton accessions, i.e., Hai7124 (Hu et al., 2019) and Pima90 (Ma et al., 2021), were generated. In addition, resequencing of multiple accessions has provided insight into the diversity and domestication of this species (Fang et al., 2017a,^2021^; Yuan et al., 2021), laying the foundation for precise genetic mapping of agronomic traits.

Research into fiber domestication has leveraged these next-generation resources, leading to the identification of key QTLs and/or genes, particularly for fiber strength, whose identities varied with the materials and techniques used. Originally, association mapping was used to identify 41 simple sequence repeat (SSR) markers associated with fiber strength of sea island cotton (Abdullaev et al., 2017). Subsequently, the first genetic map was constructed based on genotyping-by-sequencing (GBS) on a recombinant inbred line (RIL) population of *G. barbadense*, which identified 3 fiber-strength QTLs (Fan et al., 2018). In 2020, two independent genome-wide association study (GWAS) analyses were released, using different materials. The first evaluated a broad sampling of 279 sea island cotton accessions using 6303 SNPs from the CottonSNP80K array and found 11 fiber strength QTLs and an E3 ubiquitin-protein ligase gene (*GB_A03G0335*) correlated with other fiber properties (Su et al., 2020). The second GWAS analysis focused on 70 *G. barbadense* cultivars developed in Xinjiang, finding 5 fiber strength QTLs in this targeted study (Nie et al., 2020). In 2021, 240 *G. barbadense* accessions were resequenced for GWAS, which uncovered three fiber-strength candidate genes on chromosome D11 (Yu et al., 2021), while a separate GWAS analysis of 229 different sea island cotton accessions revealed 3 additional candidate loci related to fiber strength, this time on chromosome A03, and a single interspecific introgression that could increase yield but decrease fiber qualities (Fang et al., 2021). In recently published research, we conducted GWAS analyses on 336 sea island cotton accessions over multiple years and locations to identify 615 significant SNP markers and 3,032 genes related to fiber strength (FS), and a casein kinase 1-like gene (*GbFS1*) was verified (Zhao et al., 2021).

While the foregoing research has provided insight into the genetic underpinnings of fiber, these often rely on relatively large populations. Breeding methods often leverage systematic selection and hybridization through multiple generations, leading to the complexity that may be obscured in population and/or biparental cross-based analyses. In this respect, pedigree analysis may provide a more nuanced view into genetic changes selected during multi-step breeding and improvement, providing additional perspective on the cumulative changes that lead to phenotypic differences. For example, the pedigree analysis has been successfully used to increase understanding of selection for agronomic traits (including fiber quality) in Upland cotton (*Gossypium hirsutum*) (Lu et al., 2019; Ma et al., 2019). Ma et al. (2019) analyzed a *G. hirsutum* pedigree comprising seven elite and 19 backbone parents, where they characterized the pedigree, finding reduced genetic diversity accompanied by strong linkage disequilibrium and extensive selective sweeps (Ma et al., 2019). Similarly, Lu et al. (2019) resequenced a family of accessions related to *cv* CRI-12, including its parents and progenies, to reveal genes correlated with *Verticillium wilt* (118 genes), salinity (126 genes), and drought tolerance (176 genes), and documented haplotype block inheritance under artificial selection (Lu et al., 2019). In our recent paper, 178 genes related to fiber strength were screened using identity-by-descent (IBD) analysis in the *G. barbadense* XH39 pedigree, which was validated by GWAS in 336 sea island cotton accessions (Zhao et al., 2021). These studies provided a reliable method and direction for exploring pedigree breeding and trait improvement of sea island cotton.

Despite this progress in understanding fiber improvement in sea island cotton, there remain numerous questions regarding the genetics underlying phenotypic changes exhibited by modern cultivars. Xinjiang is an ideal regional model to study the improvement of sea island cotton because most cultivars were bred from a few introduced varieties, making the Xinjiang Sea Island cotton population more like a large pedigree population. In such a pedigree-like population, genetic diversity is relatively low, and unique genetic variations are more likely to be associated with the improvement of vital agronomy traits. Here, we chose a simple-kinship pedigree and the high-yielding and high-quality elite cultivar XH35 to explore key genetic factors simultaneously influencing Xinjiang Sea Island cotton quality and yield. Candidate genes identified by inheritance within the pedigree were validated by genome-wide association studies. These genes were further screened by expression analyses, narrowing to one key candidate gene, *GbCML7*, which encodes a calmodulin-like protein. The function of this key candidate gene was confirmed by analyzing the effects of haplotype transform on critical quality and yield traits. Our results provide genetic clues for fiber quality and yield co-improvement in elite sea island cotton breeding.

## Materials and Methods

### Pedigree, Planting, and Phenotyping

We used 19 accessions in a pedigree from 336 *G. barbadense* accessions (Zhao et al., 2021), maintained at China Agricultural University, Beijing. The original parent (9122И) was introduced from Turkmenistan (the former Soviet Union) in 1959; additional accessions include Giza70 (an Egyptian variety) and other 17 accessions bred in Xinjiang (Supplementary Table 1). There are seven main cultivars, including JH1, XH3, XH16, XH47, XH24, XH35, and XH39.

Phenotyping of five fiber quality traits (fiber length, FL; fiber strength, FS; fiber micronaire, FM; fiber uniformity, FU; and fiber elongation, FE) was performed in three locations over 5 years [Awat in 2018–2019, Baotou Lake (20 km away from the downtown of Kolar) in 2019, Korla in 2013, 2015, 2016, 2018, 2019], and phenotyping of five yield traits (fruiting branch number, FBN; boll number, BN; single boll weight, SBW; lint percentage, LP; seed index, SI) was completed for three locations over 6 years (one more environment than the former, namely, Korla in 2014; except that SI was obtained in Awat in 2018–2019, Baotou Lake in 2019, Korla in 2016, 2018, 2019). All accessions were planted in replicated (2×) design, with plots of 18–20 plants per row (2 m row length), ∼11 cm between plants within each row, and 66 cm between rows. Cotton was sown in mid-to-late April and was harvested in mid-to-late October in Xinjiang.

Twenty naturally opened bolls were hand-harvested to calculate the SBW (g) and gin the fiber. SI was obtained after counting and weighing 100 cotton seeds. Fiber samples were separately weighed to calculate LP. Fiber samples were evaluated for quality traits with a high-volume instrument (HFT9000) at the Ministry of Agriculture Cotton Quality Supervision, Inspection and Testing Center in China Colored Cotton Group Corporation, Ürümqi, China. Data were collected on the fiber upper-half mean length (FL, mm), FS (cN⋅tex^–1^), FM, FE (%), and FU (%).

### Genome Sequencing and Analyses

Genome information of 19 pedigree accessions was included in our 336 Sea Island accessions population (Zhao et al., 2021). They were sequenced using Illumina HiSeq PE150. The reference genome was *G. barbadense* 3–79^1^ (Wang et al., 2018). High-quality SNPs [depth ≥ 4, quality ≥ 20, the missing ratio of samples within the population ≤ of 10%, and minor allele frequency (MAF) > 0.05] were used in the phylogenetic tree, PCA, and structure analyses, whereas SNPs with a missing ratio ≤ of 20% were used in the other analyses. SNP annotation was performed according to the *G. barbadense* reference genome using the package ANNOVAR v1.0.0 (Wang et al., 2010).

### Low Diversity Block Analysis

We used sliding windows (1 Mb) and the calculation of SNP ratios to determine low diversity regions (Jiao et al., 2012; Fang et al., 2017b; Ma et al., 2019). Nucleotide diversity (θπ) was calculated from single nucleotide polymorphisms (SNPs) using VCFtools v0.1.115 (Danecek et al., 2011). Low diversity blocks were defined as segments having the lowest 5% of windowed π values (0.0000427 in our pedigree).

### Phylogenetic, PCA, and Structure Analyses

An NJ (neighbor-joining) tree was constructed using P distance in TreeBestv1.9.2 software^2^. Bootstrap values were derived from 1000 resampling. Genetic structure was assessed using the software Admixture (1.23). PCA is conducted using GCTA 1.24.2^3^ software (Li and Durbin, 2010).

### Genetic Transmission Analysis

To detect genetically transmitted regions in a pedigree, we calculated the SNP ratio between parental accessions and XH35, also called “IBD” (identity by descent) analysis. A window size of 200 SNPs, with a step size of 20 SNPs, was used to perform genomic scans (Jiao et al., 2012; Fang et al., 2017b). A window with the same SNP ratio ≥ 99% was considered as an inheritable fragment in the pedigree (Ma et al., 2019).

### Gene Identification

The genomic positions of specific fragments from low diversity blocks analysis and genetic transmission analysis were searched in Cotton Functional Genomics Database (CottonFGD^4^) (Zhu et al., 2017) for genes contained within those blocks (Supplementary Tables 5, 7).

### Genome-Wide Association Study Mapping

In our previous paper (Zhao et al., 2021), GWAS data of these ten traits were calculated by the mixed-linear model using software GEMMA 0.94.1 (genome-wide efficient mixed-model association^5^) (Zhou and Stephens, 2012). Here, we used this result to validate the genes for specific traits.

### Differential Expression Analyses

A pair of sea island cotton FS extreme cultivars, XH58 (with high fiber strength) and Ashi (with low fiber strength), were planted in the field in 2019. Bolls were collected during the initiation stage (0 DPA), cell-elongation stage (5, 10, and 15 DPA), and secondary-wall synthesis stage (20 and 25 DPA). Fibers were isolated from the boll in liquid nitrogen. Total RNA was extracted from the fibers of the boll samples with an EASYspin RNA Plant Mini Kit (Cat # RN0902, Aidlab Biotechnologies, Ltd.). The qualified RNA treated with DNase I (Takara) was used for constructing cDNA library, HiSeq sequencing, assembling, mapping [HISAT 2.0.4 (Kim et al., 2015), with default parameters], analyzing gene expression [HTSeq v0.6.1 (Anders et al., 2015), -m union], detecting SNP [GATK v3.5 (McKenna et al., 2010), QUAL < 30.0 && QD < 5.0), identifying differentially expressed genes (DESeq 1.10.1 (Anders and Huber, 2010), padj < 0.05], GO [GOSeq (Young et al., 2010), Release2.12, Corrected *P*-value < 0.05] and KEGG [KOBAS v2.0, Corrected *P*-value < 0.05 (Mao et al., 2005)] annotation according to the method in our laboratory (Guo et al., 2015; Shi G. et al., 2015; Zhao et al., 2021). The criterion of differentially expressed genes was as follows: *P*-value < 0.05 and |log_2_(fold change)| > 1.

### Co-expression Analysis

The co-expression network is constructed based on the correlation level between gene pairs among 69 expressed FS-related genes (82 in total). First, we calculated the correlation coefficient by using the ‘‘psych’’ package in R. The co-expression network was conducted using Cytoscape software v.3.8.2^6^ (Shannon et al., 2003). HUB genes were calculated using the cytoHubba plugin (Chin et al., 2014), and top ranked genes were selected from a consensus of 12 methods, including MCC, DMNC, MNC, Degree, EPC, BottleNeck, EcCentricity, Closeness, Radiality, Betweenness, Stress Clustering, and Coefficient.

### Functional Annotation and Enrichment Analysis

The original annotations files [*G. barbadense* (AD_2_) ‘3–79’ genome HAU_v2_a1], including GO annotation, KEGG annotation, and gene model (.gff3), were downloaded from COTTONGEN database^7^ (Yu et al., 2014). GO and KEGG enrichment analysis was performed using the “clusterProfiler” package in R.

### Interactive Network Analysis

Fiber-quality-related genes interacting with *GbCML7* were predicted based on their homologous genes in *Arabidopsis* using STRING V11.5^8^ (Szklarczyk et al., 2019). First, all genes identified by pedigree genetic transmission analysis and GWAS validation were aligned with *Arabidopsis* NR data using blastn^9^, with the one that scored the highest in terms of identity and coverage chosen as the homolog of that sea island cotton gene. Then, those *Arabidopsis* homologs were put into STRING to predict the interaction network. The confidence of interaction score was set to 0.400 (medium confidence) for FS-related genes and 0.700 (high confidence) for fiber-quality-related genes.

### Haplotype Analysis

*GbCML7* and genes interacting with it were subjected to SNP calling (Vcftools 0.1.16, Danecek et al., 2011) in their gene region and the promoter region (2,000 bp upstream of the gene) for the 19 pedigree accessions and a larger population of 336 sea island cotton accessions. In gene regions, only large-effect SNPs, including non-synonymous, stop-gain, and stop-loss SNPs, were further considered as haplotypes of that gene, whereas all SNPs were considered within the promoter region. Finally, only the two main haplotypes that appeared in most of the 19/336 accessions were used to analyze their effect on the phenotype. For the two *GbCML7* interacting genes, at most four haplotype combinations were used for phenotype comparison.

## Results

### Genetic Basis for Trait Improvement in Pedigree Breeding

**FIGURE 1:**
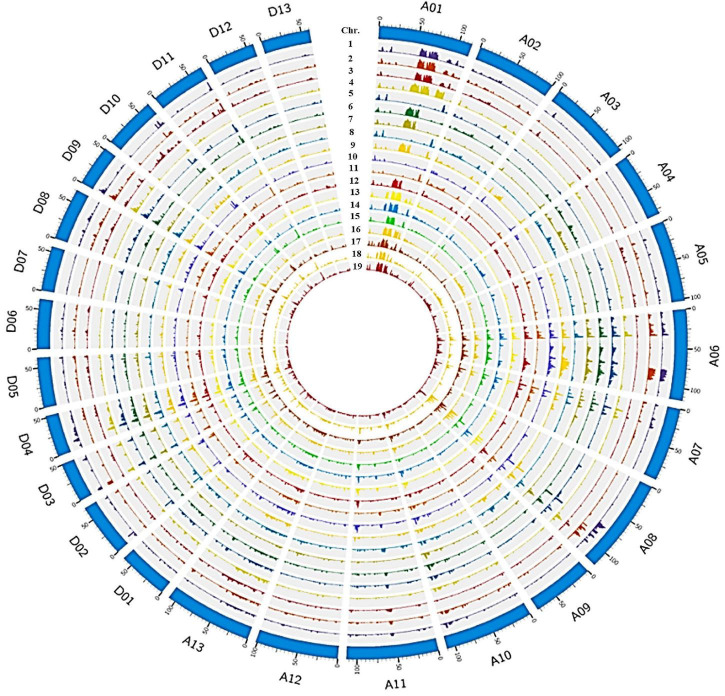
Genome wide SNP distribution across 26 chromosomes of 19 Sea Island cotton accessions in the JH1-derived pedigree. The outermost layer represents the 26 chromosomes (A01–13 to D01–13) in a clockwise direction, then from the second outside circle inward, the layers show SNP density in 1_XH39, 2_XH47, 3_Giza70-2, 4_9122И, 5_AK86430, 6_XK107, 7_JH1, 8_XH3, 9_XH6, 10_XH8, 11_XH10, 12_XH12, 13_XH20, 14_XH24, 15_XH26, 16_XH35, 17_XH16, 18_XH60, and 19_S03.

To understand the effects of pedigree-based breeding on the underlying genetics in elite *G. barbadense* lines, we resequenced 19 Sea Island cotton varieties comprising an intact pedigree (including 7 primary cultivars; Supplementary Figure 1) and built a variation map (Figure 1), generated from an average of 11.34× coverage, with ∼93.6% of the reference genome covered by at least 4× depth (Supplementary Table 1). The number of SNPs detected per sample varied from 1.0 to 1.6 M (average 1.3 M), indicating reasonable diversity among these samples. SNP density averaged 0.61 SNP⋅kb^–1^, with a range from 0.48 to 0.73 SNP⋅kb^–1^ among these related accessions, with an average of 1.66 kb between adjacent SNPs (Supplementary Table 2).

As expected, most SNPs (1.2 M) were found in intergenic regions, with far fewer found in upstream (32,257), downstream (25,362), intron (50,001), exon (24,595) regions, 5′UTR/3′UTR (4,118/6,497) or splicing sites (155; Supplementary Figure 2A and Supplementary Table 3). On average, each sample showed 15,053 non-synonymous SNPs, 8,969 synonymous SNPs, 387 stop-gain SNPs, and 57 stop-loss SNPs (Supplementary Figure 2B and Supplementary Table 3); the number of heterozygous SNPs (442343) is about half of that of homozygous SNPs (868605), accounting for 33.04% of the total SNPs (Supplementary Figure 2C and Supplementary Table 3). In total, we detected 35,426 non-synonymous SNPs, 19,994 synonymous SNPs, 931 stop-gain SNPs, and 138 stop-loss SNPs that altogether affected 17,369 genes (Supplementary Figure 2B and Supplementary Table 4). These genes are likely to play a diverse role in trait improvement during pedigree breeding.

We identified 107 low diversity segments, i.e., regions including the lowest 5% of the windowed *pi* values (Supplementary Table 5). The largest segment (∼7 Mb) was located on chromosome D11 and comprised up to 10.25% of the entire chromosome, while the shortest was ∼1 Mb on chromosomes A02/A10/D09 (Supplementary Table 5). In total, these regions covered 29 Mb in the At subgenome, and 78 Mb in Dt, which encompass a total of 3,418 genes, 952 in At and 2,466 in Dt (Supplementary Table 5). The low diversity in these regions is suggestive of purifying selection during selective breeding in this pedigree, and the asymmetric distribution between subgenomes suggests that the traits targeted by breeders were more frequently located on the Dt chromosomes.

**FIGURE 2:**
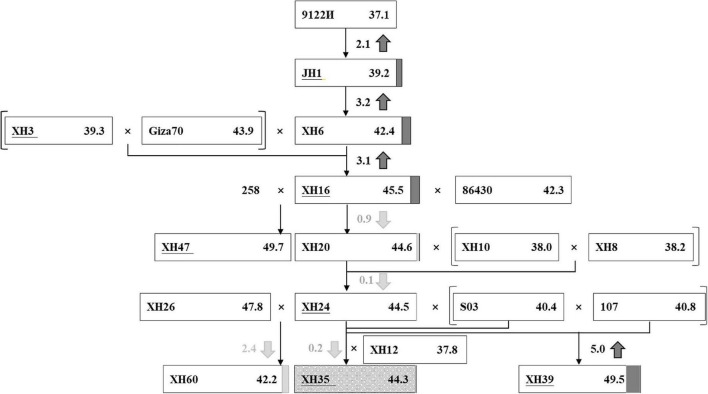
The improvement of fiber strength in a selected pedigree. Each rectangle represents an accession. In the rectangle, the accession names are written on the left and the phenotype values of fiber strength (cN-tex^−1^) are listed on the right. The different value of fiber strength between the current accession and the former parent is highlighted in the middle, with the up/down arrow showing the increase (in dark gray)/decrease (in light gray). The increment and decrement are also noted right behind the rectangle with dark gray box and light gray box, respectively. XH35, serving as the target cultivar of our research, is marked with dark gray snowflake background.

Using breeding records and the resequencing data, we traced the parental origin and genetic composition of three modern elite cultivars, XH35, XH39, and XH60 (Figure 2), focusing on XH35 as the key target of our study. The XH35-bred pedigree was initiated from the introduction of *G. barbadense* accession 9122И (FS = 37.1 cN⋅tex^–1^) from Turkmenistan, formerly of the Soviet Union, in 1959. This accession was considered a good candidate as it was suitable for the poor photothermic conditions in Tarim, Xinjiang (Supplementary Table 1). From this accession, Xinjiang breeders selected an earlier-maturity variant, JH1, with improved fiber strength (FS = 39.2 cN⋅tex^–1^), as a primary cultivar in 1967 (Figure 2). By 1988, breeders had developed a new cultivar, XH6, which further improved fiber strength (FS = 42.4 cN⋅tex^–1^) relative to the previous accessions. Fiber strength was again increased (FS = 45.5 cN⋅tex^–1^) in the next accession, XH16, which derived from hybridization between XH6 and an Egypt-introduced variety, Giza70 (Figure 2).

Due to the challenges of simultaneously improving fiber quality and yield, gains in fiber quality may lead to stagnation or reduction in measurements of fiber yield. Accordingly, after XH16, the breeding focus shifted toward yield traits, including fruit branch number (FBN), boll number (BN), boll weight (BW), *etc*., with the introduction and hybridization of several other breeding lines (Figure 2). These subsequent hybridizations and breeding efforts gave rise to three elite cultivars, XH35, XH39, and, XH60. Cultivar XH39 is characterized by longer and stronger fiber than the other accessions, whereas XH60 excels in high lint percentage and boll number; only XH35 combines fine quality (i.e., higher fiber strength) and high yield (i.e., more bolls; Supplementary Table 6).

We analyzed the improvement of these target traits, i.e., fiber strength (FS), fruit branch number (FBN), and single boll weight (SBW; Supplementary Figure 3), in the context of the pedigree for these accessions. Phylogenetics, principal component analysis (PCA), and STRUCTURE analysis suggest two subpopulations for these 19 sea island cotton accessions (hereafter called A and B, Supplementary Figures 3A–C), which reflect their history of breeding. Although most share common ancestry (aside from the foreign material used for hybridization), there became a clear division among accessions, beginning with the breeding of the first major Xinjiang cultivar, JH1. While most of the varieties in the A population are early-introduced parents, it is notable that STRUCTURE analysis of these accessions suggests that the originally introduced cultivar, 9122И, is represented by variation found in both the A and B subpopulations. Breeding of XH6, the precursor to XH16, introduced variation from foreign material (Giza70), which solely contains B-like variation. Subsequently, these later accessions began to include more B-like variation until most of the recently-selected elite cultivars, including XH35, XH39, XH60, are represented entirely by B-like variation. Notably, while the original 9122И cultivar had both A- and B-like variations, both foreign, introduced accessions, i.e., Giza70 (with relatively better fiber quality, especially fiber strength) and S03 (with relatively better yield advantages, especially boll number), contained B-like variation. Phenotypic comparisons of five fiber-quality and five yield-component traits among accessions in two subpopulations (Supplementary Figures 3D–F, 4) show that FS, FBN, and SBW were all significantly higher in B-subpopulation varieties than in A-subpopulation varieties, despite the early bias toward A-like variation. In agreement with that, correlation analysis of five fiber-quality and five yield-component traits revealed a positive and significant relationship between fiber-quality trait (FS) and yield-component factor (FBN and SBW) (Supplementary Figure 5) in these accessions, reflecting the shift toward co-improvement for FS and yield in this pedigree.

### Pedigree Analysis Identified Fiber-Strength-Related Genes via Genome-Wide Association Study

**FIGURE 3:**
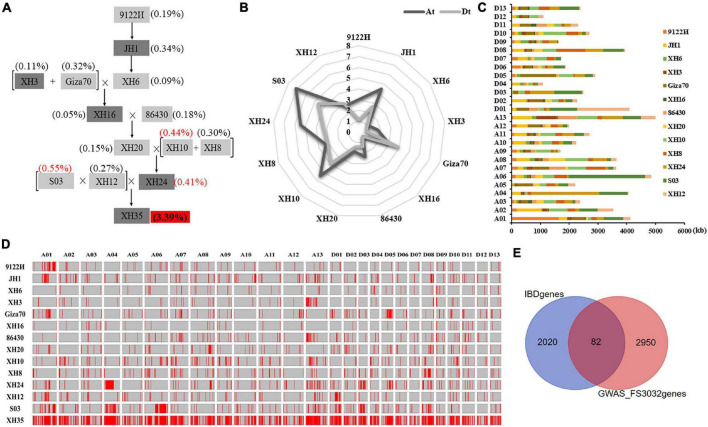
Genetics underlying fiber strength improvement in XH35-bred pedigree. **(A)** XH35-bred pedigree, including 12 parental varieties. **(B)** Total length of genomic fragments inherited from 12 parents in the At and Dt subgenome. The length of genetic components in At and Dt is indicated in dark and light gray, respectively. **(C)** Length distribution of genomic fragments inherited from 12 parents on 26 chromosomes. **(D)** Homologous fragments of XH35 in 12 related parents from the pedigree [listed on the vertical axis (left)]. The horizontal axis (top) indicates different chromosomes. Unique genetic segments in each parent are specifically passed to XH60 according to the genetic pathway shown on **(A)**. **(E)** Venn diagram showing the overlapping FS genes (in middle garnet) obtained by genetic transmission analysis and GWAS analysis.

Pedigree analysis in XH35 successfully traced 3.39% of the genomic sequences to the 13 ancestors evaluated (Figure 3A). Notably, SNPs from three parents (i.e., S03, XH10, and XH24) occupied the largest fraction of ancestor-identified SNPs in XH35 (11,731,534 bp and 0.55%, 9,351,609 bp and 0.44%, and 8,770,784 bp and 0.41%, respectively). In total, we found 72,612,359 SNPs attributed to 13 ancestors, of which 38,797,196 (53.43%) were contained within 2,104 genes. Interestingly, for most parents, the total amount of sequence traceable to a given ancestor was generally greater in the At subgenome than in Dt, with the exception of sequences traced to Giza70 and XH12 (Figure 3B). With respect to chromosomes, the inherited fragments (traceable to the 13 parents) were greatest on A13 (5,006 kb), A06 (4,846 kb), and A01 (4,124 kb; Figures 3C,D and Supplementary Table 7).

To determine the possible phenotypic effects of these SNPs, we cross-referenced the SNPs with those identified for agronomically important traits using GWAS (Zhao et al., 2021). For fiber strength, one of the initial targets of this breeding program, we found 82 FS-related genes (Figure 3E) in common between our pedigree analysis and GWAS of these same samples. In addition, we found numerous genes for other agronomic traits that were present in both our pedigree and GWAS analyses, including fiber length (FL, 31 genes), elongation rate (FE, 72 genes), uniformity (FU, 39 genes), micronaire value (FM, 84 genes), fruit branch number (FBN, 73 genes), boll number (BN, 22), single boll weight (SBW, 30 genes), lint percentage (LP, 74 genes), and seed index (SI, 43 genes; Supplementary Figure 6 and Supplementary Table 8).

### Expression Analysis Reveals a Calmodulin-Like Gene Enhancing Fiber Strength

**FIGURE 4:**
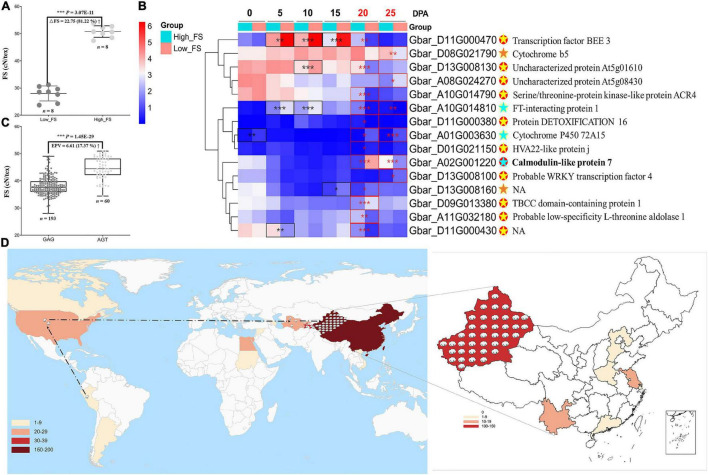
Expression analysis on the fiber strength candidate gene and geographical distribution of elite haplotype of *GbCML7*. **(A)** Phenotypic difference in fiber strength in FS extreme accessions used for expression analysis. Each solid point represents a set of original data. *n* = 8. The middle line represents the mean value, and the upper and the lower lines represent plus or minus standard deviation. High-FS and low-FS are the two FS extreme accessions, XH58 and Ashi, used for RNA-seq analysis. **(B)** Heatmap of 15 fiber-strength DEGs in 20 and 25 DPA fiber of FS extreme lines. The box color (from blue to red) represents –log_2_(FPKM + 1) value. The group in light blue and red represent accessions with high and low fiber strength, respectively. Eleven genes, marked with red solid spots, are hub genes. The remaining 4 genes are marked with yellow solid spots. Significance differences in gene expression between high and low fiber-strength accessions were analyzed with a two tailed *t*-test. *, **, *** indicates significant difference at *P* < 0.05, 0.01, 0.001, respectively. Significant differences at 20 and 25 DPA fiber are highlighted in red box. For hub genes with red spots, the light-yellow star denotes significant DEG at one stage, while the light blue denotes significance at two stages. For those genes with yellow spots, significant DEG in one stage is marked with an orange star. Gene annotations are on the far right. **(C)** Fiber strength in accessions with the two main haplotypes of *GbCML7* (*Gbar_A02G001220*). *n* represents the number of accessions with that specific haplotype. Each point represents one accession. Accessions with haplotype GAG are in dark gray, and those with haplotype AGT are in light gray. Boxes span the first to third quartiles, center lines represent median values, and whiskers show the minimum and maximum. **(D)** Geographical distribution of elite haplotype of *GbCML7*. The color gradients in the lower left corner represent the numbers of Sea Island accessions from different countries we collected. Cotton icons indicate the locations of accessions with elite haplotype *GbCML7.* The dotted line marks the origin and domestication course of sea island cotton.

To further understand the roles of these genes in controlling fiber strength, we performed differential and co-expression analyses on the 82 fiber-strength-related genes identified by the intersection of our pedigree analysis with GWAS for those same lines (Supplementary Figures 7A,B). We used RNA-seq from two extreme FS accessions (Figure 4A) at six developmental timepoints, i.e., 0, 5, 10, 15, 20, and 25 DPA (days post anthesis), to characterize the expression of these genes in the context of fiber strength. We combined these results with GO and KEGG enrichment for these 82 genes to add perspective regarding their functional classification and annotation information (Supplementary Figures 7C,D). These analyses identified 15 candidate genes that are both co-expression hub genes and significantly different in expression between FS extreme accessions in 20 and/or 25 DPA fibers (Figure 4B and Supplementary Table 9), timepoints within the critical period for secondary cell wall thickening (20–40 DPA) (Haigler et al., 2012) which likely influence fiber strength. From those 15 candidate genes, the calmodulin-like gene, *GbCML7* (*Gbar_A02G001220*), exhibited extreme differential expression in both 20 and 25 DPA fibers but no other timepoints (Figure 4A and Supplementary Table 9), potentially indicating a fiber-strength specific role for this gene in influencing secondary cell wall thickening.

Structural analysis of *GbCML7* reveals three exons (Supplementary Figure 8A), the first of which encodes four EF-hand calcium-binding domains (Supplementary Figure 8B). These result in four calcium-binding regions in the protein three-dimensional conformation (four green points represent four calcium ions, Supplementary Figure 8C), and result in a primary molecular function of calcium ion binding, as also indicated GO analysis (Supplementary Figure 8D). In addition to the extreme differences in expression, we also detected a non-synonymous mutation (G to A in sense strand) that results in an amino acid shift from glycine (G) to arginine (R) in the second EF-hand calcium-binding domain of exon 1 (Supplementary Figures 8A,B). This modification alone could affect calcium binding and fiber strength in turn. Furthermore, two additional mutations were found in the third exon: (1) a non-synonymous SNP (A to G in sense strand) that changes the arginine (R) into glycine (G), and (2) a stop-gain SNP (G to T in sense strand) that glutamate (E) used to be (Supplementary Figure 8A). These variations will greatly influence the normal function of the protein.

We evaluated the two key haplotypes, i.e., GAG and AGT, in a population context and compared the association between each haplotype and fiber strength. From a large natural population, we found 193 and 60 accessions containing the GAG and AGT haplotypes, respectively. Compared to accessions with the GAG haplotype, those with the AGT haplotype exhibited a 6.61 cN⋅tex^–1^ (17.37%) increase in fiber strength (Figure 4C). The geographic distribution of accessions with this elite FS haplotype (i.e., AGT) are found throughout the origin, domestication, and improvement route of sea island cotton as it originated in South America (Peru), moved through North America (United States domestication) and Central Asia (Tajikistan, Turkmenistan, and Uzbekistan), to China (Xinjiang, improvement) where it was incorporated into the elite lines of this region (Figure 4D).

Notably, our pedigree shows that the haplotype of *GbCML7* (GAG) in the initial ancestral parent, 9122И, is the non-elite haplotype, which is congruent with 9122И being the FS-lowest accession in our pedigree (Supplementary Figure 9). This haplotype was eventually replaced by the elite AGT-allele in XH24 (Supplementary Figure 9) where it was maintained in the subsequently-bred cultivars, XH60, XH35, and XH39 (Supplementary Figure 9). In a word, the haplotype dynamics of *GbCML7* provide insight into FS improvement in our pedigree.

### *GbCML7* Interacts With *GbTUBA3* to Synergistically Enhance Fiber Strength

**FIGURE 5:**
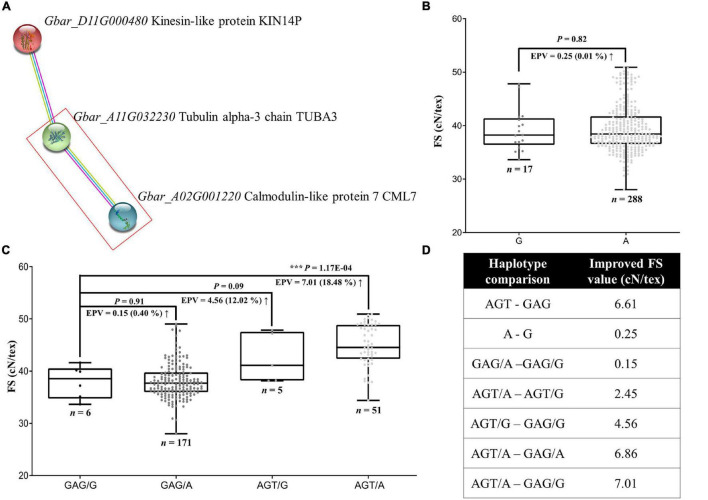
Gene interaction between FS-related genes, *GbCML7* and *GbTUBA3*, and the resulting distribution of FS. **(A)** Interaction network among three FS-relate genes, including the key candidate *GbCML7*. Colored circles represent proteins with known or predicted 3D structure inside. On the right are the names of the *G. barbadense* genes and encoded proteins. Colored lines between two circles represent the interaction relationship, where purple, blue, and yellow lines represent the source of that information, i.e., experimentally derived (purple), from curated databases (blue), and by text mining (yellow). **(B)** Fiber strength in accessions with the two main haplotypes of *GbTUBA3* (*Gbar_A11G032230*). *n* represents the number of accessions with that specific haplotype. Each point represents one accession. Accessions with the haplotype G are in dark gray and those with haplotype A are in light gray. Boxes span the first to third quartiles, center lines represent median values, and whiskers show the minimum and maximum. Significance differences in FS between the two groups of accessions with different haplotypes was analyzed using a two tailed *t*-test. *** indicates significant difference at *P* < 0.001. The up arrow indicates the increase of FS from the former to the latter. **(C)** Fiber strength in accessions with diverse interactive haplotypes between *GbCML7* and *GbTUBA3*. **(D)** Various improvement levels of FS in multiple comparisons of interaction combinations.

We considered the role of potential partners of *GbCML7* in conferring FS by leveraging gene homology and the *Arabidopsis* interaction network. Using the *Arabidopsis* homologs of our original 82 FS-related genes, we used the STRING website to search for the networks that include a homolog of *GbCML7*, which recovered a simple three-gene network composed of *Gbar_D11G000480-Gbar_A11G032230-Gbar_A02G001220* (Figure 5A), the last of which is *GbCML7*. Directly interacting with *GbCML7* is *Gbar_A11G032230*, which encodes a tubulin alpha-3 chain. *Gbar_A11G032230* interacts with *Gbar_D11G000480*, which encodes a kinesin-like protein and indirectly interacts with *GbCML7* through *Gbar_A11G03223* in the same pathway. Notably, both partners are microtubule-related genes. *Gbar_A11G032230* serves as a component of microtubules, while *Gbar_D11G000480* acts as a motivator of microtubules’ movement. This suggests that the role of *GbCML7* in conferring fiber strength may be related to microtubule generation and/or movement.

Analysis of the coding regions for these genes reveals a large-effect variation only in the gene *Gbar_A11G032230* (hereafter *GbTUBA3*), i.e., one non-synonymous mutation in the fifth exon (G to A) that results in a codon shift from GCC (alanine; A) to ACC (threonine; T). When we surveyed the effects of these haplotypes on FS, however, we observe that haplotype A (threonine) is only 0.25 cN⋅tex^–1^ (0.01%) higher than haplotype G (alanine; Figures 5B,D). This effect is relatively minor compared to the favorable haplotype of *GbCML7* (i.e., AGT), which increases FS by 6.61 cN⋅tex^–1^ irrespective of the effects of other genes (Figures 4C, 5D). Therefore, we conclude that *GbCML7* functions as a main-effect gene, whereas *GbTUBA3* works as a minor-effect gene.

Interestingly, however, there appears to be a synergistic effect when both elite haplotypes co-occur. When we analyzed FS in the context of the four combinations of *GbCML7* and *GbTUBA3* haplotypes, the additive effects of these two genes emerge (Figures 5B–D). While the advantageous allele of *GbCML7* (AGT) generally confers an increase of 6.61 cN⋅tex^–1^ in FS, the FS increase is only 4.56 cN⋅tex^–1^ in the presence of the less advantageous *GbTUBA3* allele (G). Conversely, the advantageous allele of *GbCML7* in the context of the advantageous *GbTUBA3* allele (A) confers an increase of 6.86 cN⋅tex^–1^. Likewise, the advantageous allele of *GbTUBA3* only increases FS by 0.15 when associated with the less advantageous *GbCML7* alleles, a 40% reduction from the general effect of 0.25 cN⋅tex^–1^ observed when no other genes are considered (Figures 5B–D), whereas the advantageous allele of *GbTUBA3* in the context of the beneficial haplotype of *GbCML7* results in a remarkable FS increase of 2.45 cN⋅tex^–1^, 8.8 times greater than when evaluating *GbTUBA3* alone (Figures 5B–D). Overall, the transition from the least advantageous allele combination (*GbCML7* = GAG and *GbTUBA3* = G) to the most advantageous allele combination (*GbCML7* = AGT and *GbTUBA3* = A) results in a FS increase of 7.01 cN⋅tex^–1^, which is larger than the sum of the individual effects, 6.61 and 0.25 cN⋅tex^–1^, respectively (Figures 5B–D).

Pedigree analysis of these haplotypes reveals that while the initial ancestral parent, 9122И, contains the less advantageous *GbCML7* allele (GAG), this accession is homozygous for the advantageous *GbTUBA3* allele (A; Supplementary Figure 10). The acquisition of the non-advantageous *GbTUBA3* allele (G) subsequently occurred sometime between the JH1 and XH6 breeding lines. Because no known foreign material was incorporated during this period, this allele may represent a *de novo* mutation. Heterozygosity for the non-advantageous *GbTUBA3* persisted through three stages (i.e., XH6, XH16, and XH20); however, *GbTUBA3* was returned to a homozygous advantageous state at the XH24-breeding stage, which was maintained through the modern cultivars. Conversely, most of the breeding history beginning with 9122И contained the non-advantageous allele for *GbCML7* (GAG; Supplementary Figure 10), which was only recently introduced in the 4th stage and later (starting with XH16). Strong selection for this haplotype, as well as the introduction of additional foreign material also including this haplotype, resulted in the elimination of the non-advantageous allele in recently-bred cultivars and, ultimately, to a strict co-occurrence of optimal FS alleles in modern cultivars.

### *GbCML7* Participates in the Compromised Co-improvement of Fiber Strength, Fiber Length, and Fiber Uniformity

Fiber strength (FS) is just one of many fiber quality traits breeders are interested in optimizing, but there may be trade-offs in simultaneously improving these traits. In the present analysis, we find that FS has a significant positive correlation with fiber length (FL) and fiber uniformity (FU) (Supplementary Figure 5), although this does not appear to be the result of pleiotropy. Using both our pedigree and GWAS analysis of the sample accessions, we screened the genome for traits that influenced more than one fiber trait, including FS, FL, FU, fiber elongation (FE), and fiber micronaire (FM). We recovered four genes that pleiotropically control FL and FM and 19 that pleiotropically regulate FE and FM (Supplementary Figure 11 and Supplementary Table 10), but no genes that directly influence strength and any other fiber quality trait.

**FIGURE 6:**
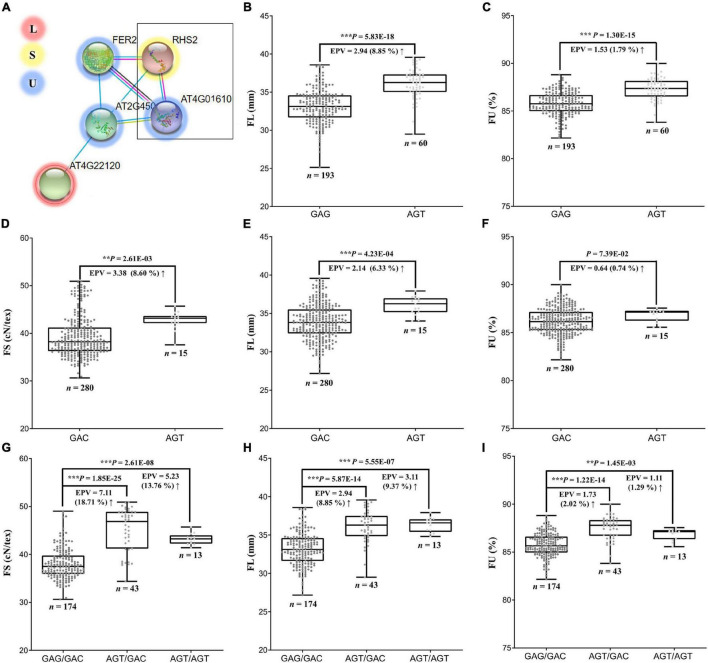
Interaction of genes related to FS, FL and FU, and their effects on phenotypes. **(A)** Interaction network among genes related to FS, FL, and FU. *Arabidopsis RHS2* is homologous to *GbCML7* and *AT4G01610* is homologous to *Gbar_D08G007940*. **(B)** FL in accessions with two main haplotypes of *GbCML7*. n represents the number of accessions with that specific haplotype. Each point represents one accession. Boxes span the first to third quartiles, center lines represent median values, and whiskers show the minimum and maximum. Significance differences in FS between the two groups of accessions with different haplotypes was analyzed using a two tailed *t*-test. **, *** indicates significant difference at *P* < 0.01, 0.001, respectively. The up arrow indicates the increase from the former to the latter. **(C)** FU in accessions with two main haplotypes of *GbCML7*. **(D)** FS in accessions with two main haplotypes of *Gbar_D08G007940*. **(E)** FL in accessions with two main haplotypes of *Gbar_D08G007940*. **(F)** FU in accessions with two main haplotypes of *Gbar_D08G007940*. **(G)** FS in accessions with the three main interacting haplotypes of *GbCML7* and *Gbar_D08G007940*. **(H)** FL in accessions with the three main interacting haplotypes of *GbCML7* and *Gbar_D08G007940*. **(I)** FU in accessions with the three main interacting haplotypes of *GbCML7* and *Gbar_D08G007940*.

We considered the possibility of genes indirectly influencing multiple pathways, such as through gene interactions. In this respect, we found that the FS candidate gene *GbCML7* may directly interact with three FU-related genes and indirectly interact with one FL-related gene (*Gbar_A05G017250*, encoding calcium-permeable stress-gated cation channel 1; Figure 6A) in the same pathway. While there were no large-effect variations (i.e., non-synonymous, stop-gain, and/or stop-loss) in the coding regions of these genes, the FU-related gene *Gbar_D08G007940* (encoding an Arginine–tRNA ligase), contains three SNPs in the promoter region (2000 bp upstream of the gene) in our pedigree accessions.

Although the FS candidate gene *GbCML7* was not identified as pleiotropic with respect to FL and/or FU, the haplotype change from GAG (non-advantageous) to AGT (elite) is associated with a 2.94 mm gain in FL and a 1.53% gain in FU (Figures 6B,C). Because *Gbar_D08G007940* was the only interacting gene to exhibit haplotype variation, we surveyed the effects of this gene on FS, FL, and FU. The transition between the GAC (non-preferred) and AGT (preferred) haplotypes of *Gbar_D08G007940* result in an average increase in FS of 3.38 cN⋅tex^–1^, in FL of 2.14 mm, and in FU of 0.64%. Interestingly, however, the effect of a double superior haplotype combination (AGT/AGT) for *GbCML7* and *Gbar_D08G007940* does not produce gains in all fiber categories. Notably, while the double superior haplotype is beneficial for FL, there appears to be an antagonistic effect for FS and FU. FS and FU for the double superior haplotype are 5.23 cN⋅tex^–1^ and 1.11%, respectively, whereas the superior-inferior combination of *GbCML7* and *Gbar_D08G007940* results in an FS of 7.11 cN⋅tex^–1^ and an FU gain of 1.73% (Figures 4C, 6). The haplotype combination of the most ancestral accession, 9122И, is GAG/GAC (i.e., double inferior), while that of the most elite line (XH35) is AGT/AGT, the double superior haplotype, indicating a possible compromise in FS and FU for gains in FL during the improvement of Xinjiang Sea Island cotton.

### *GbCML7* Raises Cotton Yield by Increasing Boll Number

**FIGURE 7:**
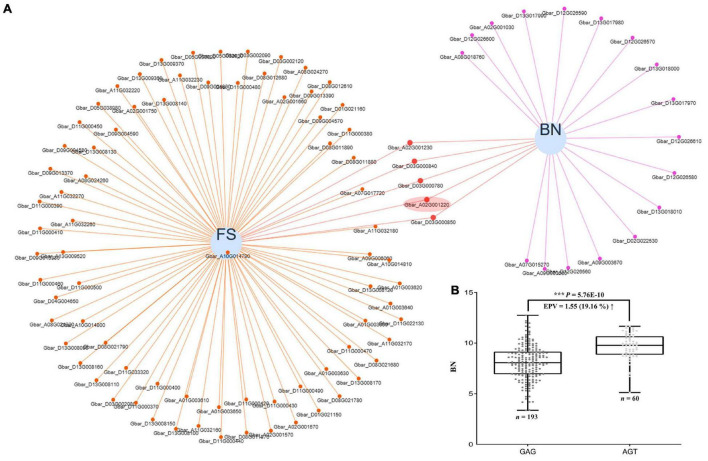
Pleiotropic genes acting on both fiber strength and boll number, and the effect of *GbCML7* haplotype shift on boll number. **(A)** Venn network showing putative pleiotropic genes for fiber strength (FS) and boll number (BN). Each blue circle represents one trait. Genes related to each specific trait are indicated as small solid spots, which are linked to the corresponding trait circle using colored lines. Small solid spots linked to two traits are regarded as putative pleiotropic genes. Fiber strength candidate gene, *GbCML7* (*Gbar_A02G001220*) is highlighted in a red ellipse. **(B)** Boll number in accessions with the two main haplotypes of *GbCML7* (*Gbar_A02G001220*). n represents the number of accessions with that specific haplotype. Each point represents one accession. Accessions with the haplotype GAG are in dark gray and those with haplotype AGT are in light gray. Boxes span the first to third quartiles, center lines represent median values, and whiskers show the minimum and maximum. *** indicates the significant difference at *P* < 0.001.

As with many agronomic traits, there is often a tradeoff in how to simultaneously improve fiber quality and yield, particularly for sea island cotton, which is characterized by superior fiber quality but low yield. Because the same genes may pleiotropically affect different phenotypes, it is useful to uncover those that have the potential to co-improve fiber quality and yield as candidates for breeding programs. In our data, we found 57 candidate genes that appear to pleiotropically influence fiber quality and yield simultaneously (Supplementary Figure 12 and Supplementary Table 11), including our FS candidate gene *GbCML7* which also contributes to boll number (BN) regulation (Figure 7A and Supplementary Table 11).

We partitioned our accessions by the two main haplotypes of *GbCML7* and found that the accessions with the FS-favorable haplotype AGT also exhibit a greater number of bolls, indicating yield improvements (Figure 7B and Supplementary Figure 13). We further examined the effects of the elite haplotype on other yield traits and discovered that accessions with the FS-favorable haplotype AGT also exhibit a larger fruit branch number (FBN), greater single boll weight (SBW), higher lint percentage (LP), and greater seed index (SI), ultimately resulting in higher lint yield (LY) and seed cotton yield (SY; Supplementary Figure 14). While seed cotton yield increased the most (23.04%), increases in both boll number (19.16%) and lint yield (18.35%) were also associated with the favorable *GbCML7* haplotype. These results indicate that this gene has a positive and comprehensive effect on many agronomically important traits in sea island cotton. Notably, the elite cultivar XH35 containing this favorable haplotype was bred for its fiber quality and yield, which pedigree analysis demonstrates is a recent addition to this pedigree.

## Discussion

### Selection From 9122И to XH35 Reveals Improved Targets of Xinjiang Sea Island Cotton

Sea Island cotton is a primary source of fine fiber worldwide. *G. barbadense* is native to NW South America, from where it spread over the last ∼7,000 years under domestication to other areas of South America, Central America, and North America (Percy and Wendel, 1990; Westengen et al., 2005; Splitstoser et al., 2016). Modern elite *G. barbadense* cultivars stem from cultivars developed in the United States, which were subsequently improved as “Egyptian cotton” and “Pima cotton” (Wendel et al., 2010), with subsequent improvement of domesticated accessions worldwide. In Xinjiang, China, many elite varieties stem from pedigree breeding of Central Asian-derived varieties, including Turkmen and Uzbek *G. barbadense* germplasm sources (Abdullaev et al., 2017), which were themselves derived from Egyptian, Egyptian-American, or/and American germplasm.

The introduction of 9122И, a variety with early maturity, from Turkmenistan in 1959, initiated what was to become the main sea island cotton varieties in the Tarim Basin, Xinjiang. In 1963, JH1 was selected from natural variants of 9122И to solve the problems of early maturity and low yield, and this accession gained in popularity to the point where it was grown in up to 249,200 hectares (He et al., 2002; Wang et al., 2002). Accession XH6 was subsequently bred from JH1 by artificial hybridization to include features such as early maturity and relatively high yield (features shared with later varieties, including XH3, XH8, XH10, and XH12). Improvement of fiber quality in these accessions gradually became the prime target of Xinjiang Island cotton breeding. An Egyptian variety, Giza70, with high fiber quality, was introduced to hybridize with XH6, generating XH16, which produces longer, stronger, finer, and more uniform fibers (Kong, 2002). From XH16 to XH20 and XH24, high yield and superior quality were further derived (Kong et al., 2018) until ultimately S03, a high-yielding variety, was added to breed a set of elite cultivars (including XH35) that integrated high yield and quality (Kong et al., 2018).

The framework of this pedigree allowed us to dissect the genetic underpinnings of improvements made during the Xinjiang breeding program. In our pedigree, we detected 107 low diversity blocks, revealing a general decrease in polymorphism (Gore et al., 2009; Lai et al., 2010), implying strong artificial selection during breeding. Therefore, although the genetic basis of the pedigreed accessions is narrow relative to other populations, the introduction of superior parents and intense directional selection for the traits of interest allowed for the simultaneous improvement of yield and fiber quality in Xinjiang Sea Island cotton.

### Genome, Transcriptome, and Phenome Collectively Identify Key Fiber-Strength Genes

Sea Island cotton has benefited from a recent expansion in genomic resources, including sequenced genomes for two accessions, i.e., 3–79 (Yuan et al., 2015) and Hai7124 (Hu et al., 2019), which were resequenced and upgraded (Wang et al., 2018; Chen et al., 2020). While these high-quality reference genomes provide a rich genetic resource for genome-wide gene mining, additional valuable information has been provided through QTL mapping in bi-/multi-parental populations and/or natural populations, which more reliably identifies genes associated with phenotypes. From these resources, several putative FS QTLs were identified. The first genetic map of sea island cotton was constructed based on 143 RILs derived from the Chinese *G. barbadense* cultivar 5,917 and the American Pima S-7, which recovered 3 QTLs (*qFS-LG21–1_A_, qFS-LG1–1_B_*, and *qFS-LG4-1_C_*) mapped to FS (Fan et al., 2018). Two years later, 11 FS QTLs (251 associated genes) were identified in 279 sea island cotton accessions, and a single pleiotropic gene, *GB_A03G0335* encoding an E3 ubiquitin-protein ligase, was found to be associated with FL, FS, FU, and FE (Su et al., 2020). Recently, 106 SNPs linked to 572 genes were significantly associated with FS, but only three of those fiber-strength candidate genes, *GB_D11G3437* (*HD16*), *GB_D11G3460* (*WDL2*), and *GB_D11G3471* (*TUBA1*), were also identified by haplotype analysis and expression validation (using transcriptome data and qRT-PCR) in a natural population (Yu et al., 2021).

The earliest pedigree study in cotton was in 2019, using a *G. hirsutum* pedigree surrounding Ekangmian 9, in which lint-percentage-related genes were identified and validated by qRT-PCR expression and haplotype analyses (Ma et al., 2019). Another paper reported on a pedigree centered on CRI-12, where some resistance genes were detected based on selective sweep and haplotype block inheritance analyses, confirmed by transcriptome data (Lu et al., 2019).

Summarizing the empirical methods obtained from the above research, we integrated pedigree genetic transmission analyses and GWAS mapping (Zhao et al., 2021) using genomic sequencing data from an elite pedigree that has produced many excellent main cultivars including XH35. Candidate genes for FS were further screened by transcriptome data from different fiber development stages. One key FS gene, *GbCML7*, was validated using haplotype analysis in a wider natural population.

### Synergistic Improvement of Multiple Fiber-Quality Traits

We identified a major-effect FS gene, *GbCML7* (encoding a calmodulin-like protein), which might interact with a minor-effect FS gene, *GbTUBA3* (encoding a tubulin alpha-3 chain), in a synergistic fashion. In addition, *GbCML7* might also interact with three FU-related genes (*Gbar_D05G031150*, encoding a ferritin-3; *Gbar_A11G008030*, encoding a metalloendoproteinase 4; *Gbar_D08G007940*, encoding an Arginine-tRNA ligase), and indirectly interact with one FL-related gene (*Gbar_A05G017250*, encoding calcium-permeable stress-gated cation channel 1) in a pathway. This is consistent with the positive correlation among FS, FL, and FU in the phenotype analysis of our pedigree.

Calmodulin-like proteins (such as GbCML7) are highly conserved Ca^2+^-binding messenger proteins in plant cells (Berridge et al., 2000) that bind Ca^2+^ ions using four EF-hand motifs (helix-loop-helix structure), which activates the calmodulin-like protein to interact with downstream target proteins (Cheval et al., 2013). As early as 1992, the influence of calcium during cotton fiber development was evaluated during primary and secondary wall development, specifically focusing on the soluble and wall-bound activities of peroxidase and *O*-diphenol oxidase in cotton fibers. Basra et al. (1992) found that the addition of calcium (1 mM) to developing cotton fibers increased the enzyme activity of peroxidase and *O*-diphenol oxidase, whereas the addition of a calcium chelator and calmodulin antagonist decreased enzyme activities, suggesting a role for calcium-calmodulin in the regulation of these enzymes through synthesis and/or secretion to the cell wall (Basra et al., 1992).

In addition to calmodulin, *GbCML7* potentially interacts with two microtube-related genes, *Gbar_D11G000480* (encoding a kinesin-like protein) and *Gbar_A11G032230* (encoding a tubulin alpha-3 chain) in a pathway. Cortical microtubules undergo dramatic reorganization during fiber development in cotton (*G. hirsutum*), and the cotton kinesin-like calmodulin-binding protein associates with cortical microtubules (Preuss et al., 2003). Calmodulin might enhance cell elongation by restraining the binding of plasma membrane polypeptides to cortical microtubules and resultant microtubule depolymerization (Yamada et al., 2015).

Additional evidence from *G. hirsutum* supports the role of calmodulins in influencing fiber development. A *GhCaM7* (calmodulin)-like gene in *G. hirsutum* exhibited high expression in elongating fibers, and its silencing resulted in shortened fiber length (Cheng et al., 2016). In *G. barbadense*, an *ATCML24* homolog cloned from accession H7124, *GbCML24*, affected fiber length in ovule culture, and many hormone response elements related to fiber development were found around 500 bp upstream of its start codon (Wang et al., 2019).

The CML genes modulate cotton fiber development based on a positive feedback regulatory pathway involving Ca^2+^, H_2_O_2_, and ROS. That is, calmodulin (CaM) increases H_2_O_2_ through a Ca^2+^/CaM-dependent NAD kinase that affects the available NADPH concentration during assembly and activation of NADPH oxidase (Harding et al., 1997; Lee et al., 1997), further modulating ROS production (Tang et al., 2014). The production of ROS may increase Ca^2+^ in the cytoplasm by activating Ca^2+^ channels and transporting Ca^2+^ into the cells or by releasing Ca^2+^ from the Ca^2+^ pool (Pariente et al., 2001; Tang et al., 2014). The increased cytoplastic Ca^2+^, in turn, activates CaM7 (Tang et al., 2014). Notably, H_2_O_2_ and ROS are positive factors in early fiber elongation, but high concentrations of H_2_O_2_ will prematurely promote secondary wall formation (Potikha et al., 1999), restricting fiber elongation; therefore, high concentrations of ROS will inhibit late fiber elongation, causing slightly shorter mature fibers (Tang et al., 2014).

### Simultaneous Enhancement of Fiber Quality and Yield by a Pleiotropic Gene

It is well known that Sea Island cotton (*G. barbadense*) has superior fiber quality but a low yield. The most efficient way to circumvent this bottleneck is to identify a large-effect, pleiotropic gene that has simultaneous positive effects on fiber quality and yield in Sea Island cotton, circumventing the need to transform *G. barbadense* with multiple yield-related genes from *G. hirsutum*, or the laborious construction of upland cotton and sea island cotton introgression lines. In our study, we found a main-effect FS key gene, *GbCML7*, which shows potential to increase production (seed cotton yield and lint yield) by impacting multiple yield traits, including fruit branch number, boll number, single boll weight, lint percentage, and seed index. While several studies have reported the role of CML genes in fiber (Basra et al., 1992; Potikha et al., 1999; Tang et al., 2014; Yamada et al., 2015; Cheng et al., 2016; Wang et al., 2019), only one has considered its simultaneous relevance to cotton yield. Notably, this study found that a *GhCaM7*-like gene had a positive effect on both cotton fiber elongation and biomass production by affecting Ca^2+^ signatures and downstream signaling pathways of CaM. When this *GhCaM7*-like gene was silenced, it resulted in both a decrease in fiber length and a reduction in multiple phenological traits, such as stem height, leaf dimensions, seed length, and 100-seed weight (Cheng et al., 2016). This provides experimental support for the pleiotropic effect of the *GbCML7* gene in fiber quality and yield. Future research will be necessary to reveal the regulatory network of *GbCML7* and its mode of action. From a practical standpoint, however, elite varieties with superior haplotypes, like XH35, can be targeted for expanded use in cotton production.

## Conclusion

We analyzed genomic variation in 19 sea island cotton accessions from a single pedigree and performed genetic transmission analysis. With the aid of GWAS mapping, we identified genes related to fiber quality and yield. A calmodulin-like gene, *GbCML7*, was screened by expression and haplotype analyses. We found that *GbCML7* might interact with a relatively minor-effect gene, *GbTUA3*, together contributing to an increase of fiber strength. Additionally, *GbCML7* may interact with genes affecting FU and FL, thereby contributing to the cooperative and simultaneous improvement of FS, FL, and FU. Importantly, we show that *GbCML7* affects cotton yield, particularly by affecting boll number. Our work provides a vital genetic factor for the co-improvement of fiber quality and yield of Sea Island cotton.

## Data Availability Statement

The original contributions presented in the study are included in the article/(Zhao et al., 2021) Supplementary Material, further inquiries can be directed to the corresponding authors.

## Author Contributions

JH and JK conceived and designed the research. JK, WW, AA, NZ, and JH prepared the population materials. KJ and NZ performed transcriptome analyses. WW, ZP, BG, JZ, MW, LX, JY, XN, HX, AA, CZ, PL, and JK performed field experiments and phenotyping. WW and NZ performed data integration. NZ performed data analyses integrally. CC selected the pedigree accessions and performed the population structure analysis. ZP, BG, DL, PL, CC, YS, NZ, and CEG contributed to the project discussion. NZ and JH prepared the manuscript. JH, CEG, and JFW revised the manuscript. All authors contributed to the article and approved the submitted version.

## Conflict of Interest

The authors declare that the research was conducted in the absence of any commercial or financial relationships that could be construed as a potential conflict of interest.

## Publisher’s Note

All claims expressed in this article are solely those of the authors and do not necessarily represent those of their affiliated organizations, or those of the publisher, the editors and the reviewers. Any product that may be evaluated in this article, or claim that may be made by its manufacturer, is not guaranteed or endorsed by the publisher.

## Footnotes

1
http://cotton.hzau.edu.cn/EN/download.php

2
http://treesoft.sourceforge.net/treebest.shtml

3
http://cnsgenomics.com/software/gcta/#PCA

4
http://www.cottonfgd.org/

5
http://www.xzlab.org/software.html

6
https://cytoscape.org

7
http://www.cottongen.org/

8
https://string-db.org/

9
https://blast.ncbi.nlm.nih.gov/
