# Supplementary material for: A Calmodulin-Like Gene (GbCML7) for Fiber Strength and Yield Improvement Identified by Resequencing Core Accessions of a Pedigree in Gossypium barbadense
Source: Front Plant Sci. 2022 Feb 3;12:815648. doi: 10.3389/fpls.2021.815648 (PMC8850914; doi:10.3389/fpls.2021.815648)
Supplement: Supplementary file 1 [file Data_Sheet_1.docx]

Supplementary Material

## Supplementary Figures


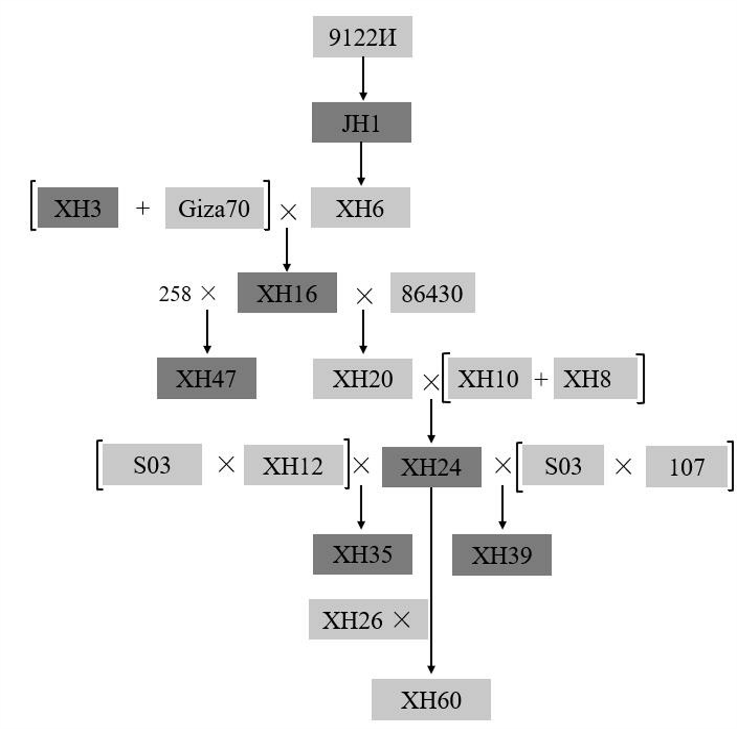


**Supplementary Figure 1.** JH1 pedigree consisting of 19 sea island cotton accessions. Main cultivars are shown on dark gray background, and other sequenced accessions are shown on light gray background.


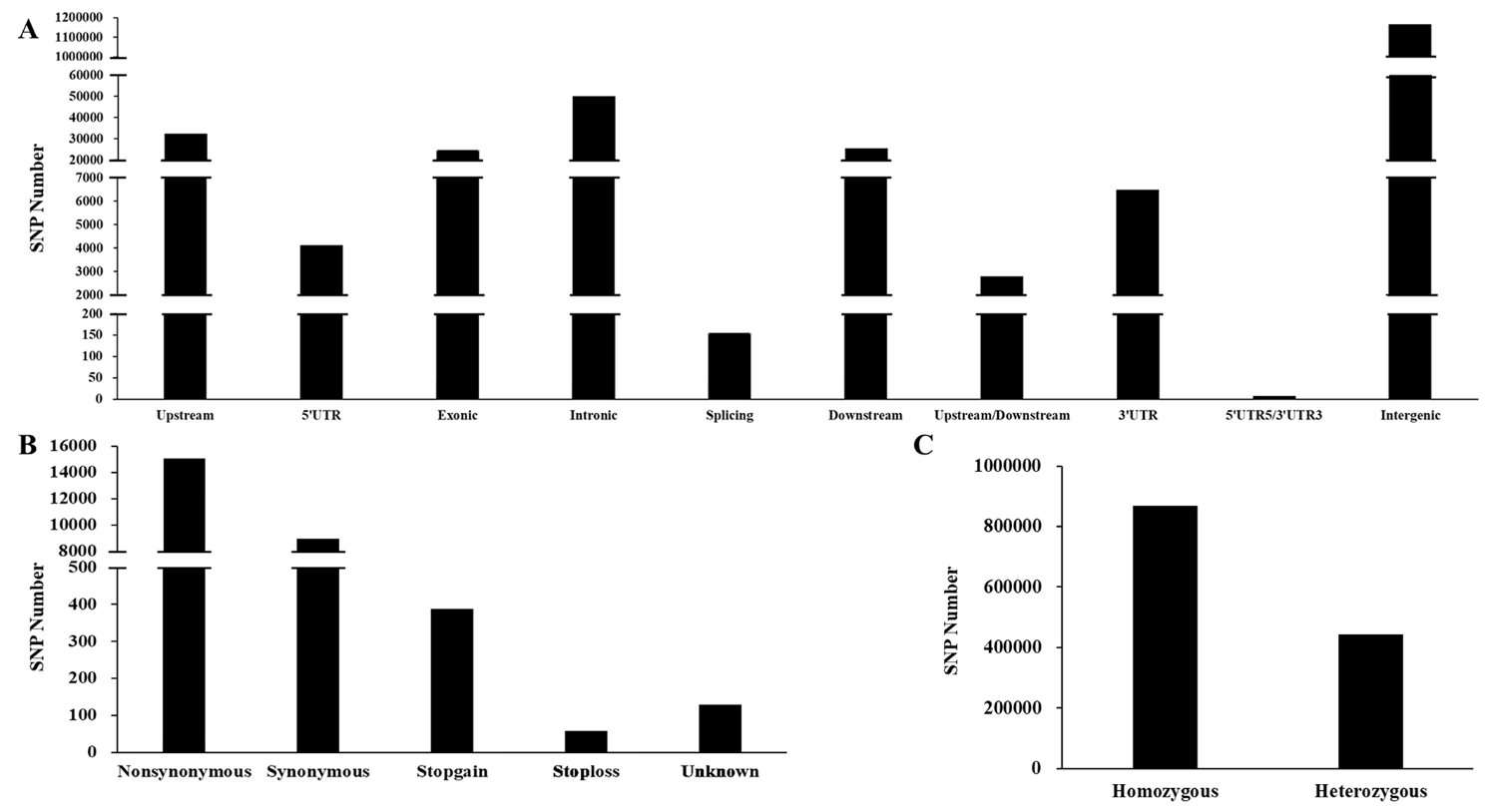


**Supplementary Figure 2.** SNP distribution across the whole genome. **(A)** SNP number parsed by genomic region. **(B)** SNP number in exons parsed by category. **(C)** The number of homozygous and heterozygous SNPs.


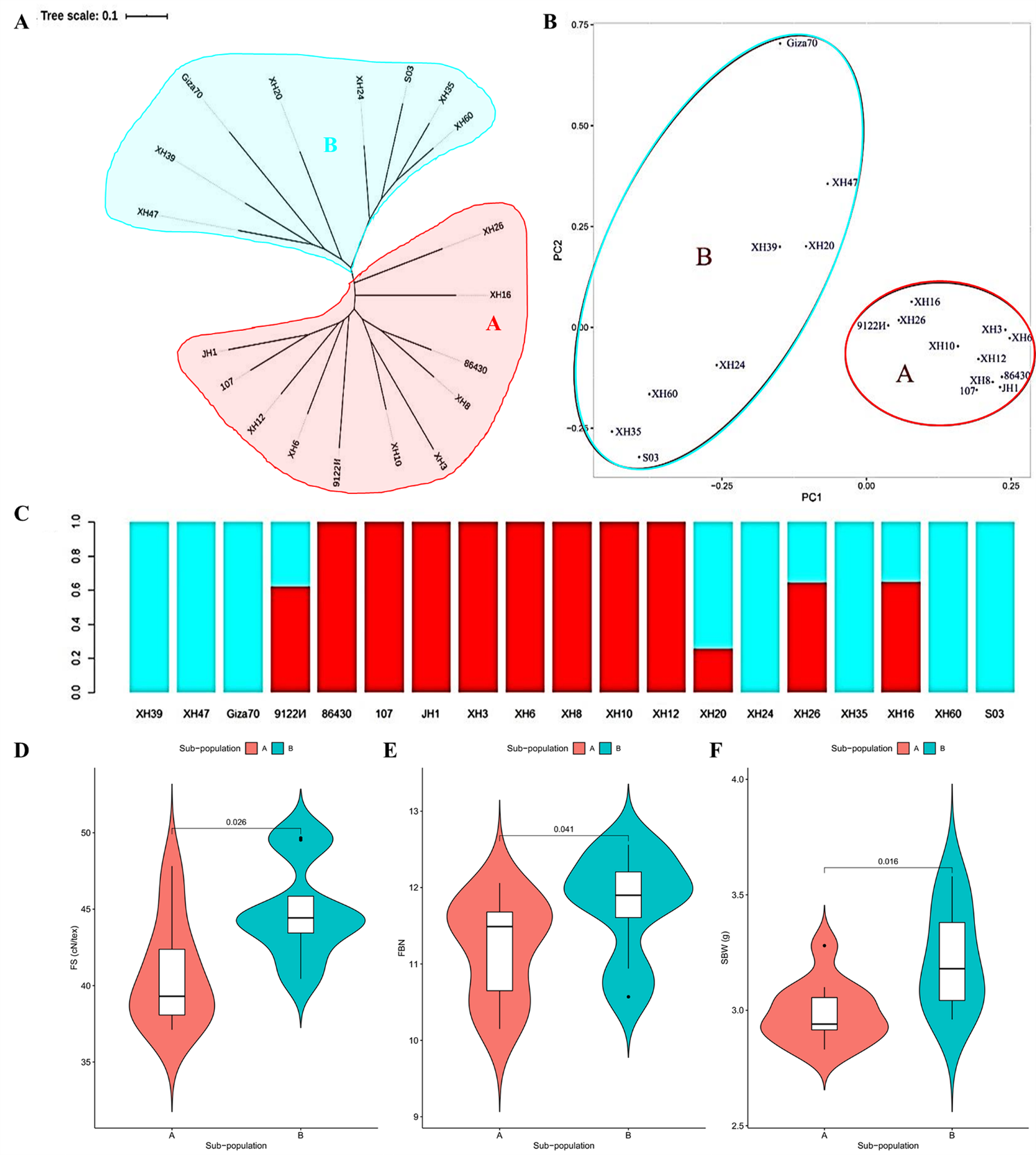


**Supplementary Figure 3.** Phylogenetic tree, PCA, structure, and phenotype divergence of 19 Sea Island cotton accessions in JH1 pedigree. **(A)** Phylogenetic tree of 19 accessions in JH1 pedigree. **(B)** PCA plot of the first two components (PC1 and PC2). **(C)** Structure analysis with K = 2. The y axis quantifies cluster membership, and the x axis represents the different accessions. **(D)** Phenotypic differences in fiber strength (FS) of two sub-populations. **(E)** Phenotypic differences in fruit branch number (FBN). f, Phenotypic differences in single boll weight (SBW). A and B represent two sub-populations resulted from phylogenetic, PCA, and structure analyses. The accessions in A (11) and B (8) sub-populations are the same for all analyses here. P-values are noted above the black lines between two violin boxes.


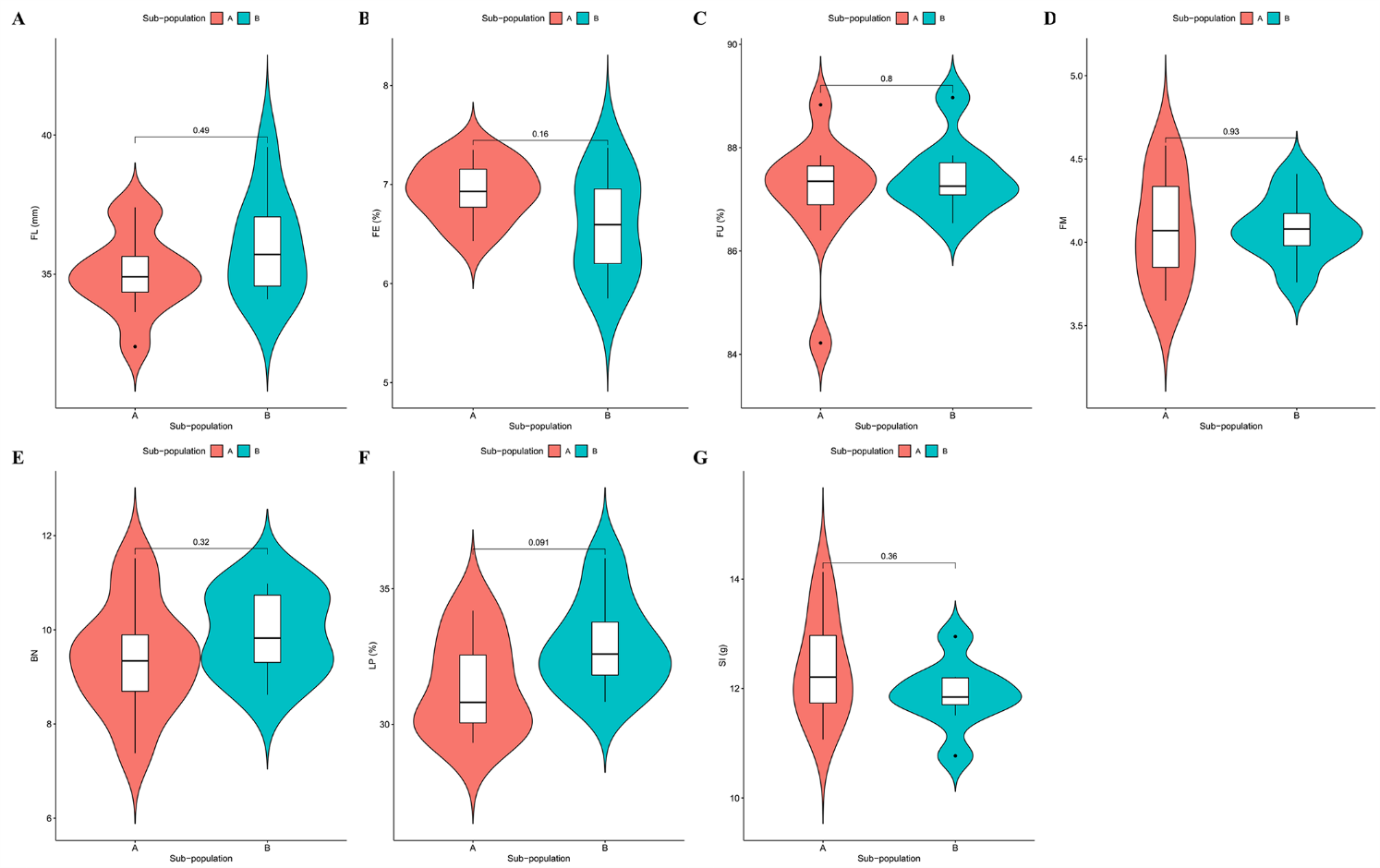


**Supplementary Figure 4.** Trait differences in two subpopulations for the other 7 traits. **(A)** Fiber length (FL, mm) in A and B subpopulations. **(B)** Fiber elongation (FE, %) in A and B subpopulations. **(C)** Fiber uniformity (FU, %) in A and B subpopulations. **(D)** Fiber micronaire (FM) in A and B subpopulations. **(E)** Boll number (BN) in A and B subpopulations. **(F)** Lint percentage (LP, %) in A and B subpopulations. **(G)** Seed index (SI) in A and B subpopulations. A and B represent two sub-populations resulting from phylogenetic, PCA, structure analyses, depicted as red and blue violins, respectively. The accessions in A (11) and B (8) sub-populations are the same for all analyses here. The significant difference between subpopulations was evaluated with two tailed t-tests. P-values are noted above the black lines between two violin boxes (red and blue). In the boxes, the center line denotes the median, then box limits are the upper and lower quartiles, and the whiskers mark the range of the data. The width of the violin represents the probability of each y-axis data.


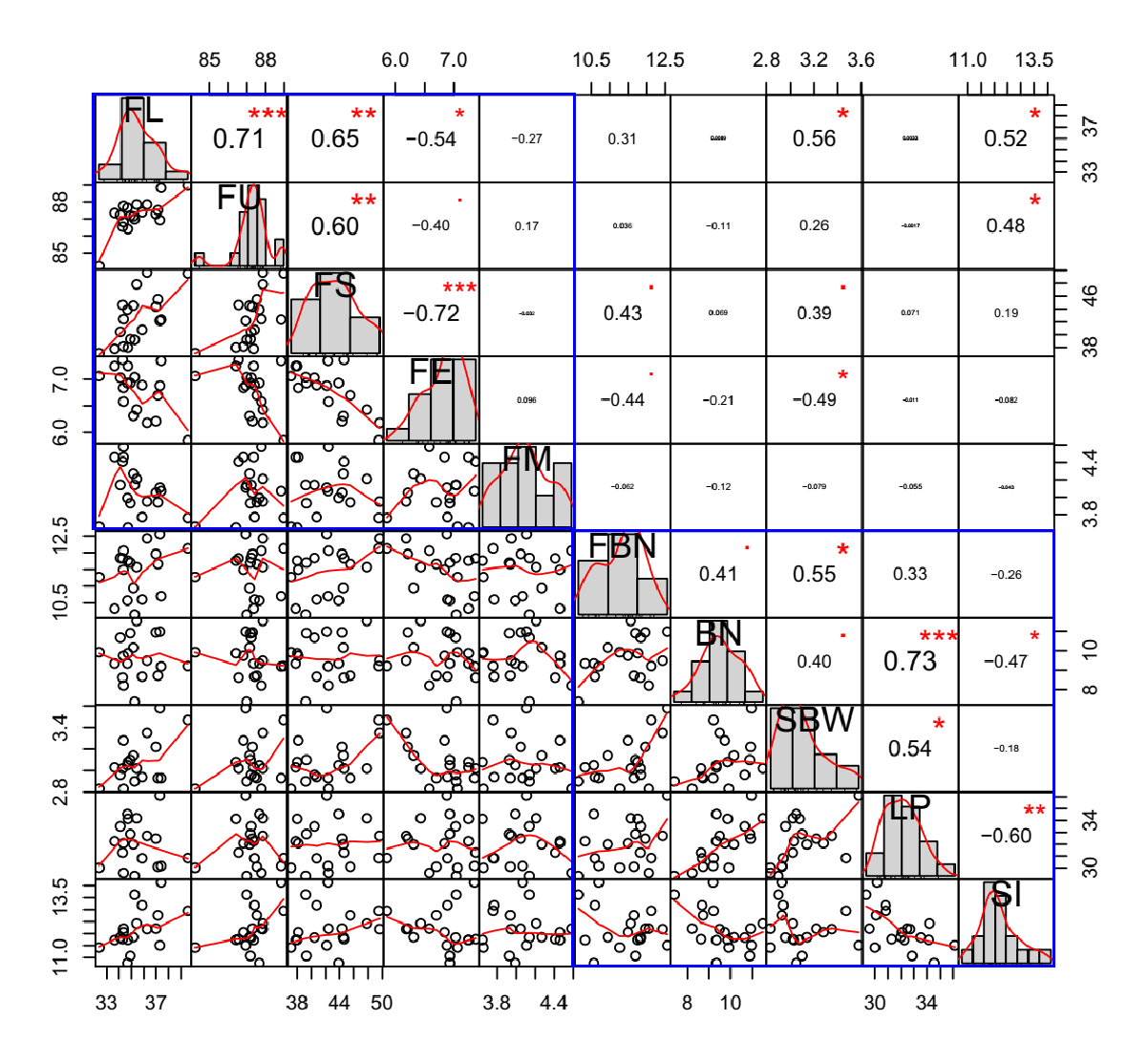


**Supplementary Figure 5.** Frequency distribution and correlation coefficients of phenotypic values for 10 traits in our pedigree composed of 19 Sea Island cotton accessions. The blue box in the upper left corner includes five fiber-quality traits, i.e., fiber length (FL, mm), fiber uniformity (FU, %), fiber strength (FS, cN·tex^-1^), fiber elongation (FE, %), and fiber micronaire (FM). The blue box in the lower right corner covers five yield components, i.e., fruit branch number (FBN), boll number (BN), single boll weight (SBW, g), lint percentage (LP, %), and seed index (SI, g). *, **, *** indicates significant difference at P< 0.05, 0.01, 0.001, respectively.


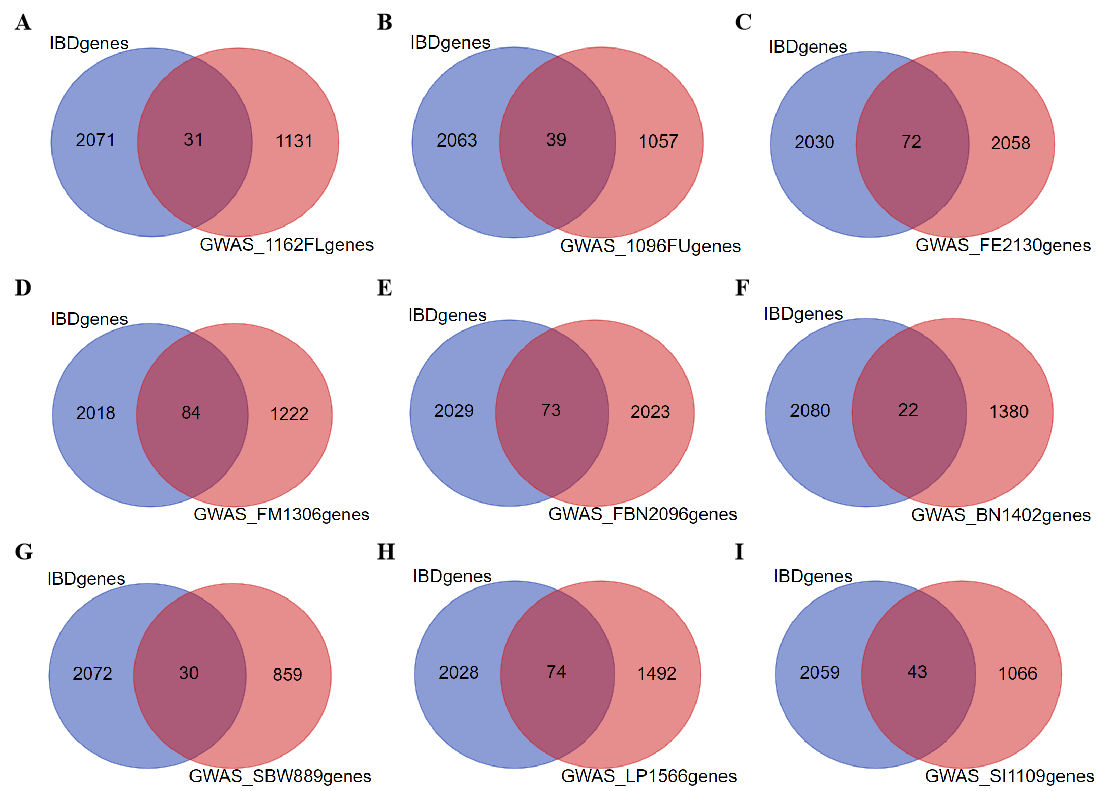


**Supplementary Figure 6.** The number of genes identified by pedigree genetic transmission analyses and validated by GWAS mapping for 9 traits. The numbers in the blue circles represent the quantity of genes discovered by pedigree genetic transmission analysis (also called IBD analysis). Meanwhile, those in red circles represent the quantity of genes mapped by GWAS analysis. **(A-I)**, Venn diagrams related to four fiber-quality traits and five yield-component factors, including fiber length (FL, **A**), fiber uniformity (FU, **B**), fiber elongation (FE, **C**), fiber micronaire (FM, **D**), fruit branch number (FBN, **E**), boll number (BN, **F**), single boll weight (SBW, **G**), lint percentage (LP, **H**), and seed index (SI, **I**), respectively.

**
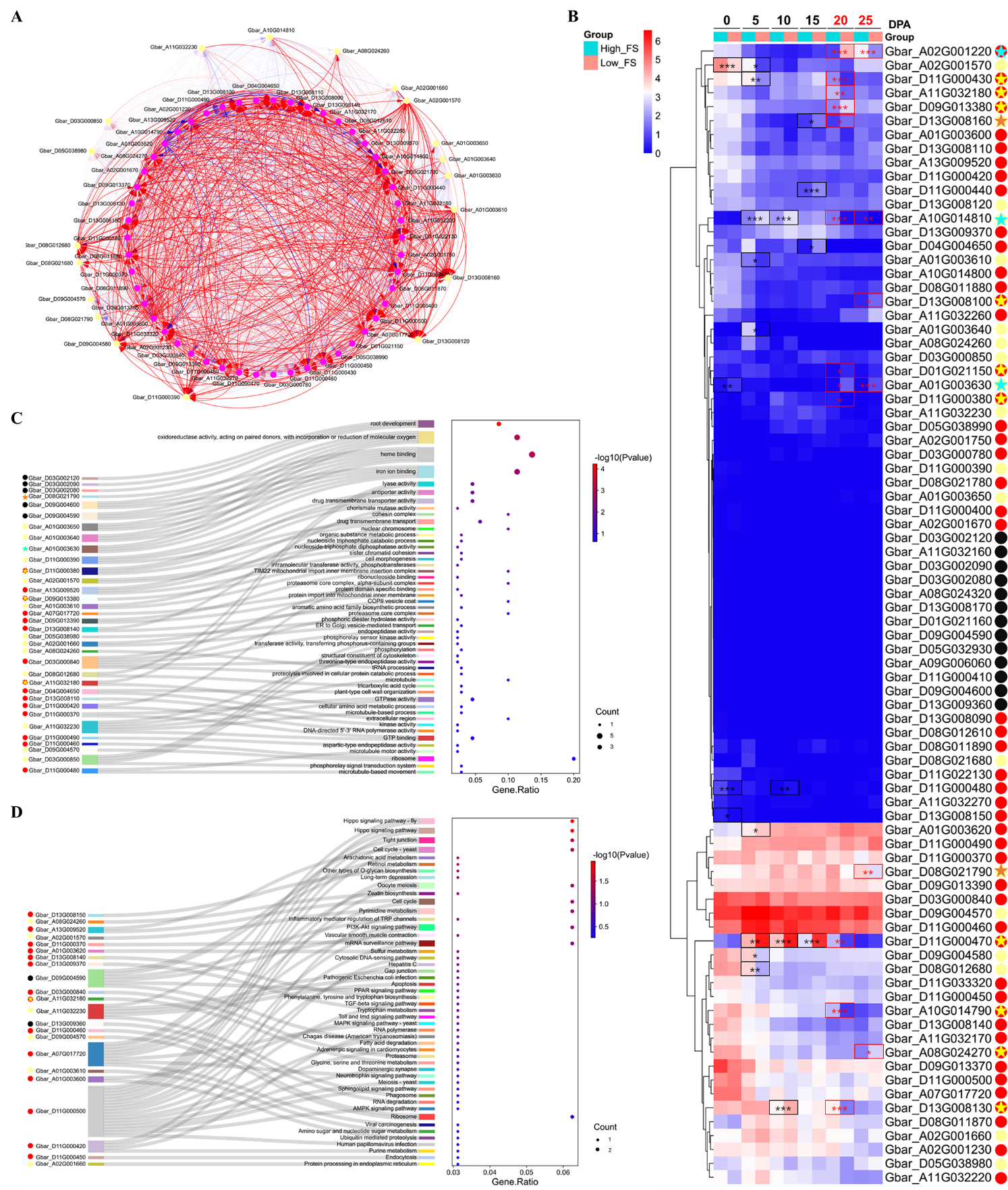
**

**Supplementary Figure 7.** Co-expression network, differential expression, and functional annotation of fiber-strength genes. **(A)** Co-expression network of correlated fiber-strength genes. Positive and negative correlations between two of 69 expressed fiber-strength genes are shown as red and blue lines, respectively. Line widths are associated with correlation strength. Transparency of lines varies from 50, 100, 250, indicating significant difference at P< 0.05, 0.01, 0.001, respectively. For significant difference at P> 0.05, line transparency is set as 10, which is barely visible to the naked eye. **(B)** The heat map of all 82 fiber-strength genes in 0, 5, 10, 15, 20, 25DPA fiber of FS extreme lines. The groups in light blue and red represent accessions with high and low fiber strength, respectively. Thirteen genes, marked with black solid spots, have no expression, and 50 genes, marked with red solid spots, are hub genes. The remaining 19 genes are marked with yellow solid spots. The significance of gene expression differences between high and low fiber-strength accessions was analyzed with two tailed t-test. *, **, *** indicates significant difference at P< 0.05, 0.01, 0.001, respectively. Significant differences in 20 and 25DPA fiber are highlighted by the red box for fiber-strength genes. The corresponding DEGs are marked with stars. For hub genes with red spots, the light-yellow stars represent those where DEG is significant at one stage, and the light blue present those with significance at two stages. For those genes with yellow spots, the significant DEG at one stage is marked with the orange star. **(C)** GO annotation of fiber-strength genes. **(D)** KEGG annotation of fiber-strength genes. the enriched number is shown as circular size. The annotation term from top to bottom is in order of significant levels (-log10Pvalue) from high (red) to low (blue).

**
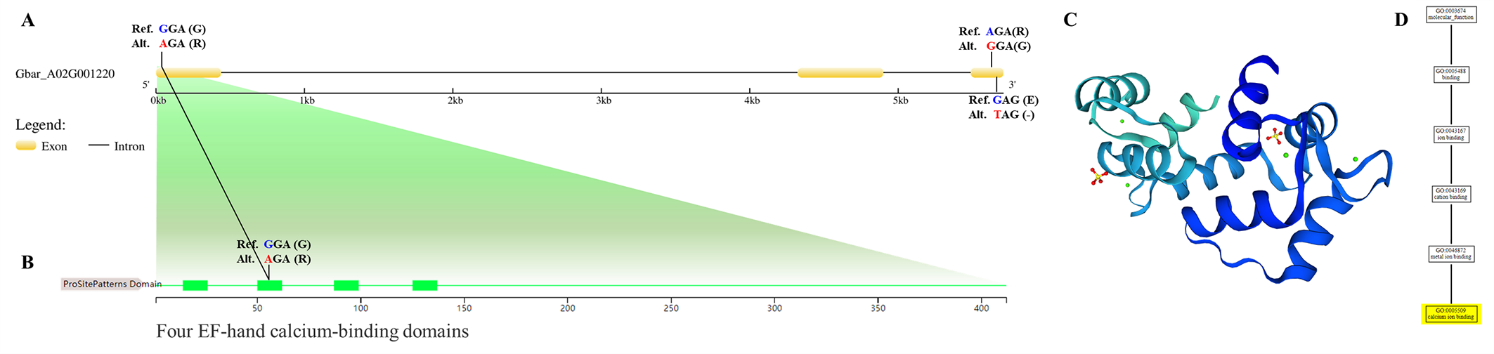
**

**Supplementary Figure 8.** Gene structure, protein domain, 3-D conformation and GO annotation of FS candidate gene, *GbCML7* (*Gbar_A02G001220*). **(A)** Gene structure of *Gbar_A02G001220*. The yellow box and black line represent exon and intron, respectively. Ref. and Alt. represent two kinds of haplotypes, where blue and red nucleotide acids are the key mutations. Three nucleic acids form a codon, and the amino acid encoded by that codon is in parentheses. **(B)** Protein domain of *Gbar_A02G001220*. Each green box represents an EF-hand calcium-binding domain. **(C)** 3-D conformation of *Gbar_A02G001220*. Each green point represents a calcium iron. **(D)** GO annotation of *Gbar_A02G001220*. Hierarchical annotation tree belongs to molecular function.


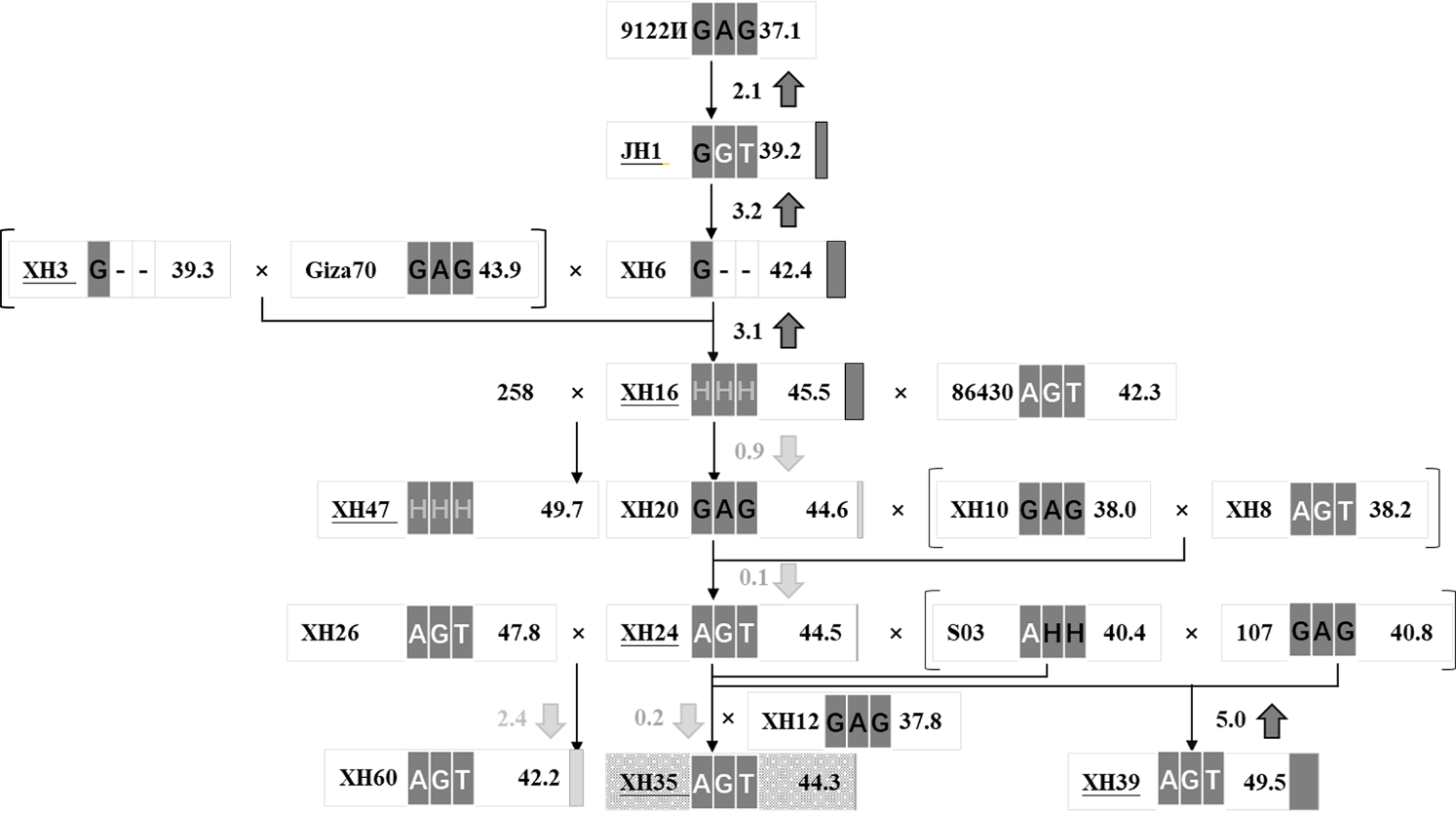


**Supplementary Figure 9.** Haplotype dynamics of FS candidate gene *Gbar_A02G001220* during the pedigree breeding process. Each rectangle represents an accession. In the middle of each accession box, there are three small dark-gray boxes that include the three large-effect SNPs inside the FS candidate gene *Gbar_A02G001220*. The favorable variation type is shown as white font, the adverse variation type is in black, and the heterozygous type is in light gray. “-” represent not detected. For more information about this figure, please refer to Figure 2.

**
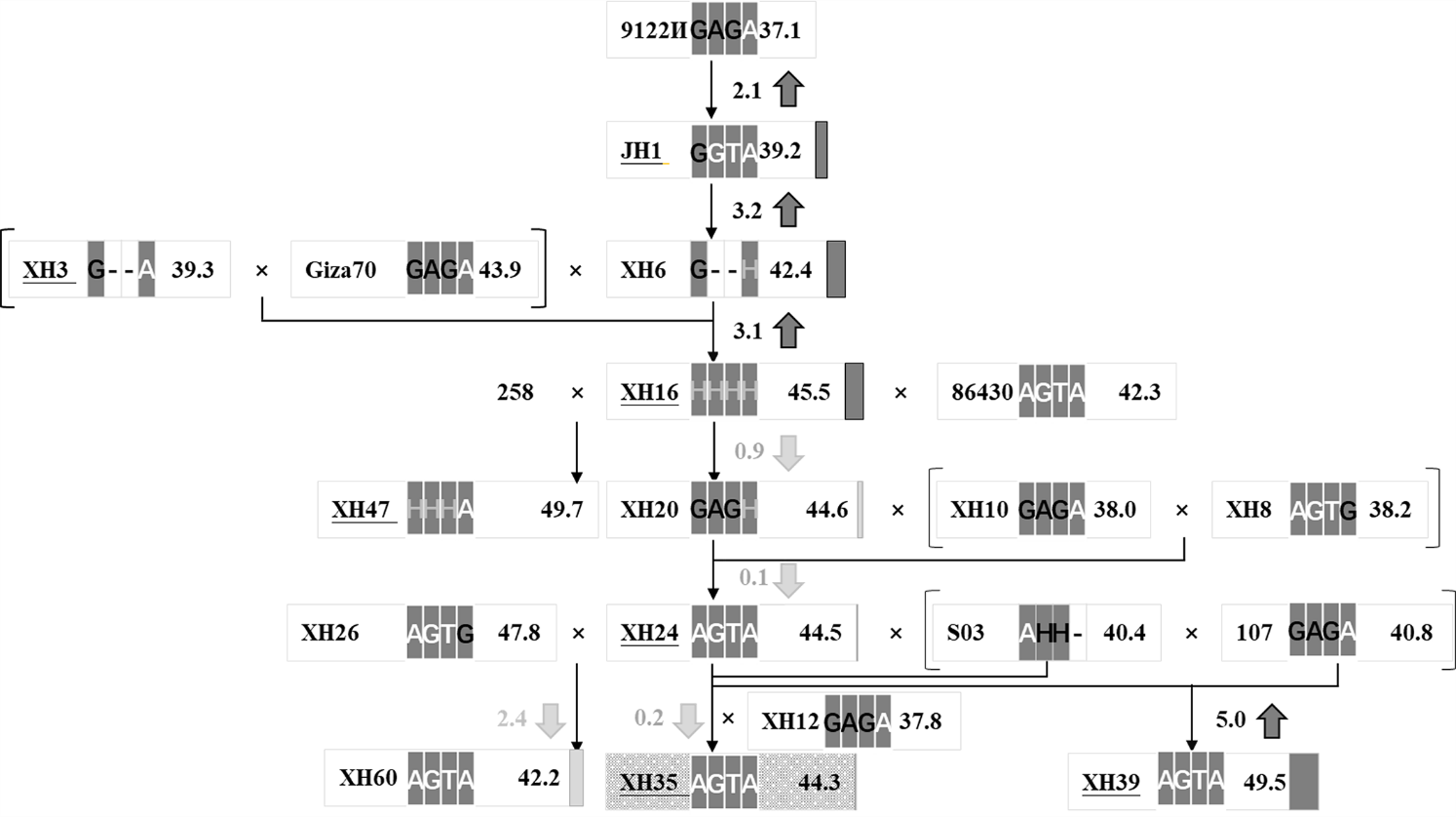
**

**Supplementary Figure 10.** Haplotype interaction of FS candidate gene *GbCML7* and *GbTUA3* during the pedigree breeding process. Each rectangle represents an accession. In the middle of each accession box, there are four small dark-gray boxes, the first three include three large-effect SNPs in the exonic region of *GbCML7* while the fourth includes one large-effect SNPs in the exonic region of *GbTUA3*. The favorable variation type is shown as white font, the adverse variation type is in black, and the heterozygous type is in light gray. “-” represent not detected. For more information about this figure, please refer to Figure 2.

**
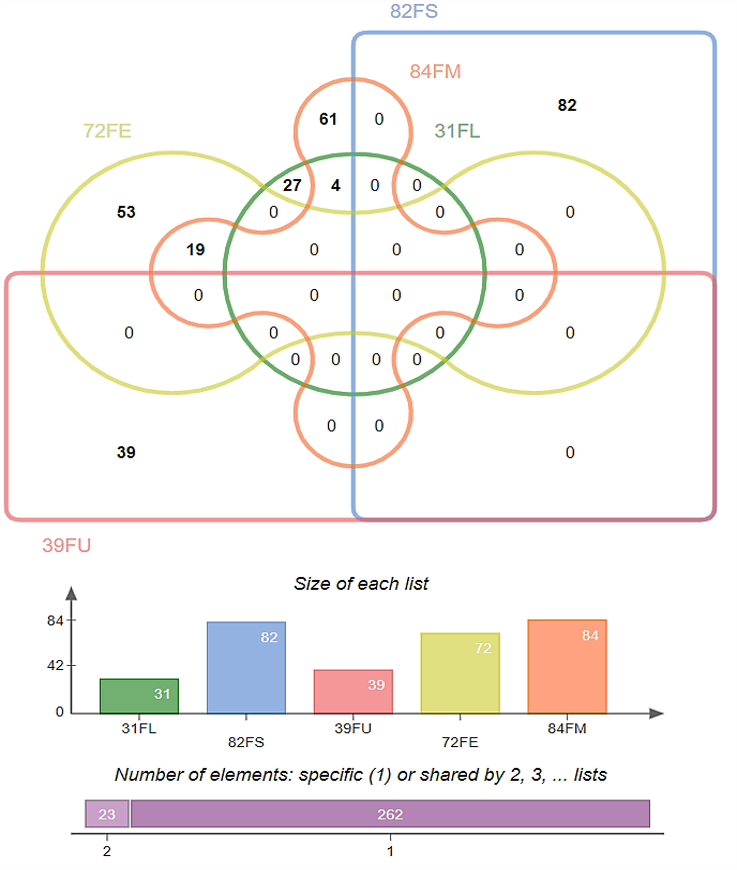
**

**Supplementary Figure 11.** Combinatorial mapping of pleiotropic genes among five fiber-quality traits. The loop curves in the upper panel in green, blue, pink, yellow, orange depict the number of genes related to FL, FS, FU, FE, and FM, respectively. Therefore, if one number is present in the overlapping region of two loop curves, it means there are the corresponding number of pleiotropic genes affecting both fiber-quality traits simultaneously. Here there are only 4 pleiotropic genes controlling FL and FM, and 19 pleiotropic genes regulating FE and FM. The bars in the middle panel are colored in green, blue, pink, yellow, and orange to represent the number of genes related to FL, FS, FU, FE, and FM, respectively. The vertical axis represents gene number and the name of traits is along the horizontal axis. The corresponding gene number for each trait has also been written inside and below the bars. The lower panel shows the number of genes specific to one trait and that which is shared by multiple traits. It is obvious that there are only 23 pleiotropic genes that are responsible for two traits, the other 262 are single-trait-related genes.

**
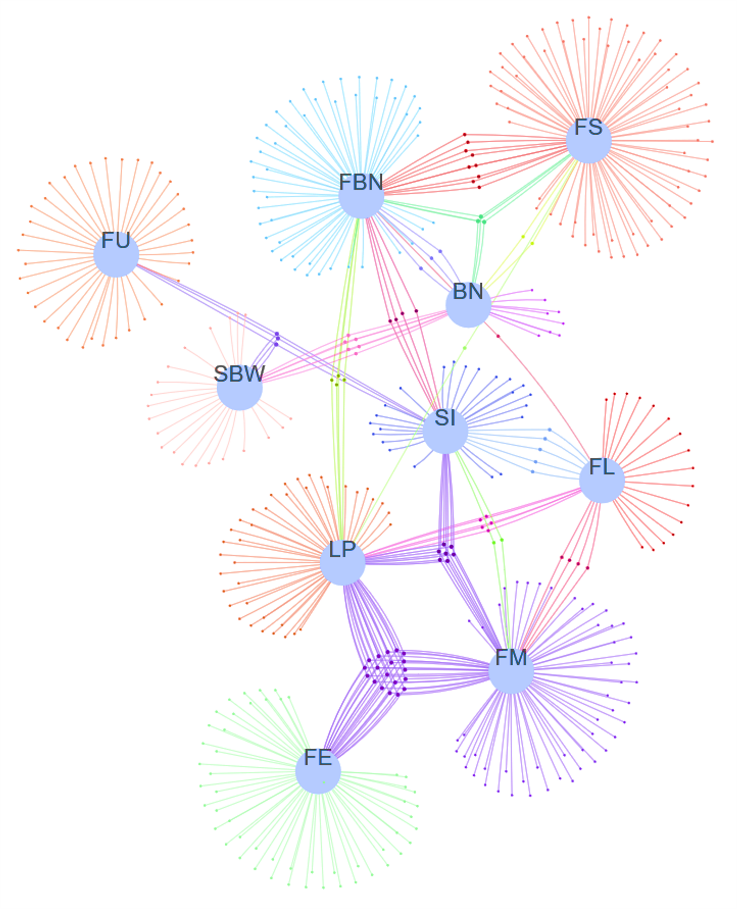
**

**Supplementary Figure 12.** Pleiotropic genes among five fiber-quality and five yield traits. Each blue circle represents one trait. Genes related to the specific trait are indicated as small solid spots, which are linked with the corresponding trait circle using colored lines. Small solid spots that are linked to at least two traits are regarded as pleiotropic genes. FL: fiber length. FU: fiber uniformity. FS: fiber strength. FE: fiber elongation. FM: fiber micronaire. FBN: fruit branch number. BN: boll number. SBW: single boll weight. LP: lint percentage. SI: seed index.

**
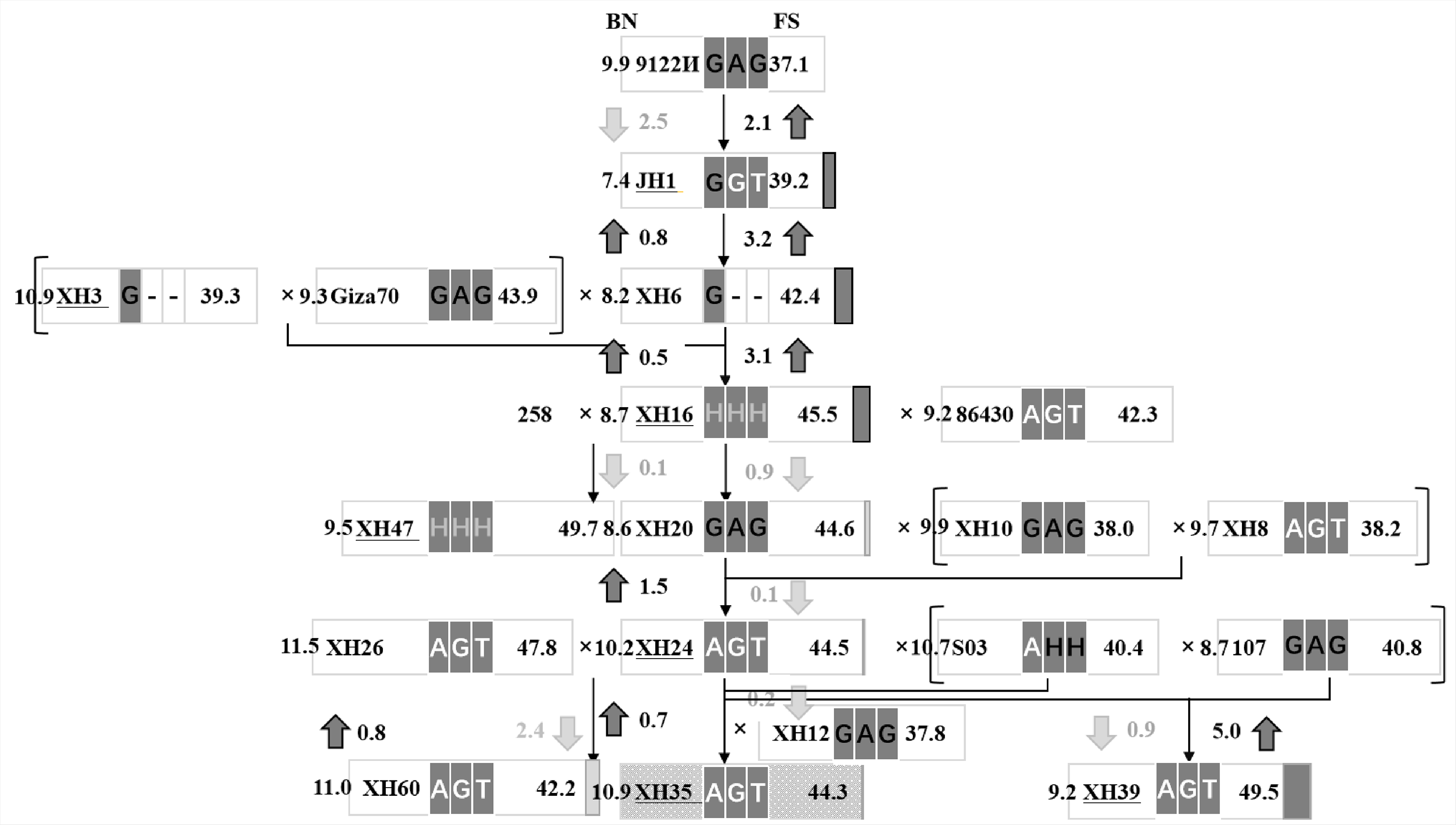
**

**Supplementary Figure 13.** Boll number dynamics controlled by FS candidate gene *Gbar_A02G001220* during the pedigree breeding process. Each rectangle represents an accession. In the middle of each accession box, there are three small dark-gray boxes that include the three large-effect SNPs inside the FS candidate gene *Gbar_A02G001220*. The favorable variation type is shown as white font, the adverse variation type is in black, and the heterozygous type is in light gray. “-” represent not detected. The phenotypic data of boll number were marked on the left side of the accession name and the change values were shown between two accessions. For more information about this figure, please refer to Figure 2.

**
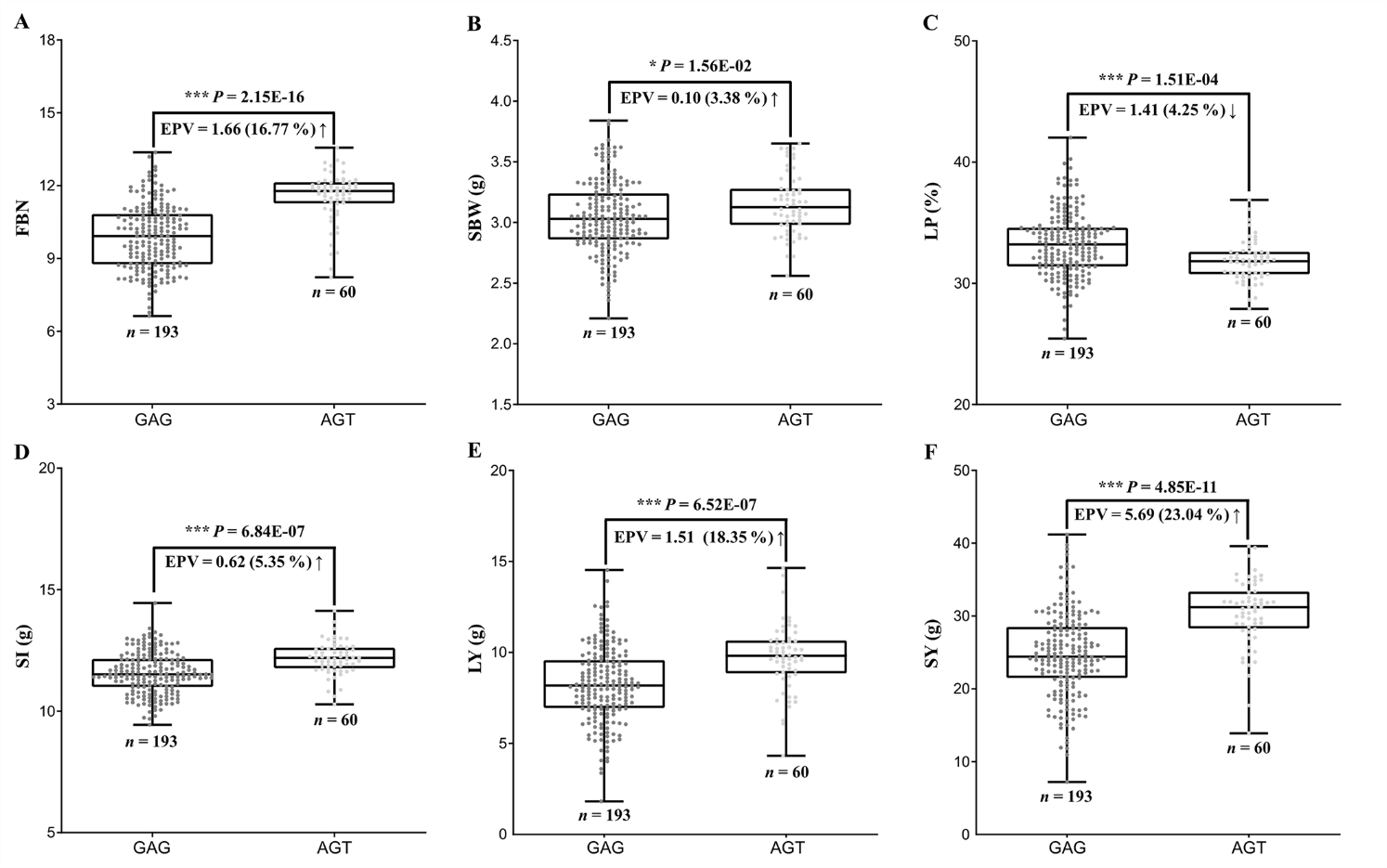
**

**Supplementary Figure 14.** Yield trait improvement resulting from haplotype shift of FS candidate gene *GbCML7*. **(A)** Fruit branch number (FBN) in accessions with two main haplotypes of *GbCML7*. n represents the number of accessions with that specific haplotype. Each point represents one accession. Accessions with haplotype GAG are in dark grey and those with haplotype AGT are in light grey. Boxes span the first to third quartiles, center lines represent median values, and whiskers show the minimum and maximum. The significant difference of FS between two kinds of accessions with different haplotypes was analyzed with two tailed t-test. *, *** indicates significant difference at P < 0.05, 0.001, respectively. The up arrow indicates the increase from the former to the latter. **(B)** Single boll weight (SBW) in accessions with two main haplotypes of *GbCML7*. **(C)** Lint percentage (LP) in accessions with two main haplotypes of *GbCML7*. **(D)** Seed index (SI) in accessions with two main haplotypes of *GbCML7*. **(E)** Lint yield (LY) in accessions with two main haplotypes of *GbCML7*. **(f)** Seed yield (SY) in accessions with two main haplotypes of *GbCML7*.
